# ZC3H18 specifically binds and activates the *BRCA1* promoter to facilitate homologous recombination in ovarian cancer

**DOI:** 10.1038/s41467-019-12610-x

**Published:** 2019-10-11

**Authors:** Arun Kanakkanthara, Catherine J. Huntoon, Xiaonan Hou, Minzhi Zhang, Ethan P. Heinzen, Daniel R. O’Brien, Ann L. Oberg, S. John Weroha, Scott H. Kaufmann, Larry M. Karnitz

**Affiliations:** 1 Division of Oncology Research, Mayo Clinic, Rochester, MN USA; 2 Department of Pharmacology, Mayo Clinic, Rochester, MN USA; 3 Division of Medical Oncology, Mayo Clinic, Rochester, MN USA; 4 Division of Biomedical Statistics and Informatics, Mayo Clinic, Rochester, MN USA

**Keywords:** Ovarian cancer, Tumour-suppressor proteins

## Abstract

Reduced BRCA1 expression causes homologous recombination (HR) repair defects in high-grade serous ovarian cancers (HGSOCs). Here, we demonstrate that *BRCA1* is transcriptionally activated by a previously unknown function of ZC3H18. We show that ZC3H18 is a DNA-binding protein that interacts with an E2F site in the *BRCA1* promoter where it facilitates recruitment of E2F4 to an adjacent E2F site to promote *BRCA1* transcription. Consistent with ZC3H18 role in activating BRCA1 expression, ZC3H18 depletion induces *BRCA1* promoter methylation, reduces BRCA1 expression, disrupts HR, and sensitizes cells to DNA crosslinkers and poly(ADP-ribose) polymerase inhibitors. Moreover, in patient-derived xenografts and primary HGSOC tumors, *ZC3H18* and *E2F4* mRNA levels are positively correlated with *BRCA1* mRNA levels, further supporting ZC3H18 role in regulating *BRCA1*. Given that *ZC3H18* lies within 16q24.2, a region with frequent copy number loss in HGSOC, these findings suggest that *ZC3H18* copy number losses could contribute to HR defects in HGSOC.

## Abstract (web summary)

High-grade serous ovarian cancers (HGSOCs) have defects in homologous recombination despite a lack of *BRCA1/2* mutations. Here, the authors show that ZC3H18 positively regulates *BRCA1* transcription and its loss causes *BRCA1* promoter methylation and increased HR deficiency in HGSOCs.

## Introduction

Homologous recombination (HR) is a high-fidelity DNA repair mechanism that requires the sequential activities of a series of proteins, including BRCA1 and BRCA2 tumor suppressors^1^. Defects in HR are a defining feature of high-grade serous ovarian cancers (HGSOCs), the most common and lethal ovarian cancer subtype^1,2^. The most frequent causes of HR defects are deleterious mutations in *BRCA1* and *BRCA2* (ref.^1^), which are associated with increased response rates to platinum-based therapies, enhanced disease-free survival, and improved overall survival^1–3^. HGSOCs with deleterious *BRCA1/2* mutations are also sensitive to poly(ADP-ribose) polymerase (PARP) inhibitors^1,2^.

Notably, many HGSOCs have HR defects despite a lack of mutations in *BRCA1/2* and other known DNA repair genes^4^. A substantial fraction of those are due to reduced *BRCA1* transcription, which is associated with HR defects in HGSOCs^5–8^. Two known mechansisms that cause reduced BRCA1 expression include (1) hypermethylation of the *BRCA1* promoter, which occurs in 8–15% of HGSOCs;^9,10,11^ and (2) mutational inactivation of CDK12 (ref.^11^), an RNA polymerase II C-terminal domain (CTD) kinase that regulates the transcription of *BRCA1* and other genes^12,13^. Additionally, *BRCA1* transcription is controlled by a complex array of transcription factors, coactivators, and corepressors that interact with the *BRCA1* promoter^14–16^. However, a complete understanding of the transcriptional regulation of *BRCA1* is lacking.

Here, we report on a previously uncharacterized mode of BRCA1 transcriptional regulation. We show that *BRCA1* transcription is regulated by ZC3H18, which we demonstrate has a previously unknown biochemical function: ZC3H18 is a DNA-binding protein that interacts with an E2F site in the *BRCA1* promoter and that activates transcripton. Accordingly, these studies expand the known roles for ZC3H18, which was previously shown to participate in RNA processing by mediating mRNA export, degradation, and transcription of a subset of protein-coding genes through its association with the mRNA cap-binding complex and the nuclear exosome-targeting complex^17–20^. This study also shows that ZC3H18 binding to an E2F site in the *BRCA1* promoter enhances the association of E2F4 with an adjacent E2F site to activate *BRCA1* transcription. Consistent with these observations, *ZC3H18* and *E2F4* mRNA levels correlated with *BRCA1* mRNA levels in primary human HGSOC tumors and patient-derived xenograft (PDX) models.

Collectively, these results discover an additional biochemical function for ZC3H18; uncover a uncharacterized mechanism of *BRCA1* transcriptional regulation; and because *ZC3H18* is located in a region (chromosome 16q24.2) of recurrent copy number loss in HGSOC^21,22^, suggest that reduced ZC3H18 levels may be an unrecognized contributor to diminished BRCA1 expression and HR defects in HGSOC.

## Results

### ZC3H18 depletion induces an HR defect and DNA damage sensitivity

Copy number losses in chromosomal region 16q24.2 are a common event in HGSOC (Supplementary Fig. 1a). Indeed, some studies have reported 16q24.2 loss to be among the most frequent copy number variation in HGSOC^21,22^, raising the possibility that genes located within this region could impact HR. To assess the potential role of genes in this region in HR, we conducted an siRNA screen of known protein-coding genes at 16q24.2 using OVCAR-8 cells that have a genomically integrated DR-GFP^23^ reporter construct^12^. Among the 16 protein-coding genes at 16q24.2, depletion of ZC3H18 had the largest effect on HR (Supplementary Fig. 1b).

**Fig. 1:**
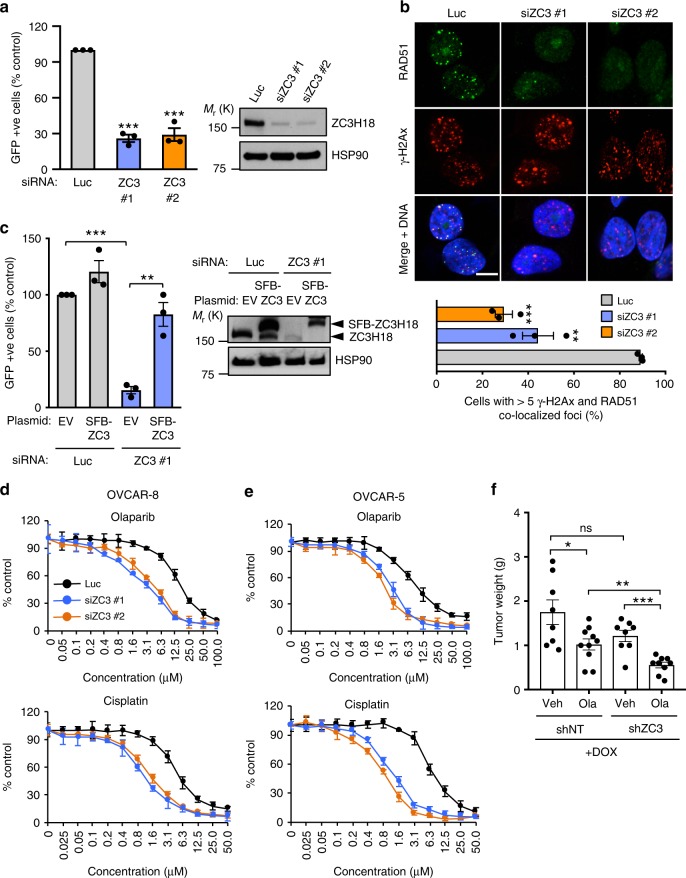
ZC3H18 depletion disrupts HR. **a** Analysis of HR efficiency. OVCAR-8-DR-GFP cells were transfected with pCβASceI plasmid and control luciferase (Luc) or independent ZC3H18 siRNAs (siZC3 #1 and siZC3 #2). Cells were analyzed for GFP fluorescence (left panel) and immunoblotted for ZC3H18 and HSP90 (right panel) 72 h later. HR efficiencies were normalized to cells with Luc siRNA. **b** OVCAR-8 cells were transfected with control Luc or ZC3H18 siRNAs and co-immunostained with RAD51 and γ-H2AX antibodies (upper panel shows representative image). Cells with ≥5 colocalized RAD51 and γ-H2AX foci were quantitated (bottom panel) 72 h later. **c** DR-GFP-OVCAR-8 cells were transfected with pCβASceI plasmid and control Luc siRNA or ZC3H18 siRNA #1 plus empty vector (EV) or siRNA-resistant ZC3H18 plasmid (SFB-ZC3H18). Cells were analyzed for GFP (left panel) and immunoblotted for ZC3H18 and HSP90 (right panel shows representative immunoblot) 48 h later. **d**, **e** OVCAR-8 (**d**) and OVCAR-5 (**e**) cells were transfected with control Luc or ZC3H18 siRNAs. 48 h later, cells were immunoblotted for ZC3H18 and HSP90 (Supplementary Fig. 2a) or were re-plated, treated with cisplatin for 3 days or olaparib for 7 days, and analyzed by MTS assay. **f** OVCAR-8 cells with stably transduced, doxorubicin-inducible non-targeting shRNA (shNT) or ZC3H18 shRNA (shZC3) were innoculated into mice. 5 days later, shRNAs were induced by feeding doxycline chow, and mice were treated with vehicle (Veh) or olaparib (Ola). After 4 weeks of treatment, tumor weights were determined. Shown are means ± SEM from three independent experiments in **a**, **b** (bottom panel), and **c**. Representative images of three independent experiments are shown in **b** (top panel).In all experiments, ≥100 cells were counted per experiment. Scale bar, 10 µm. Graphs in **d** and **e** represent one of three independent experiments that gave similar results. Error bars are standard error of triplicate wells from an individual representative experiment. Shown are means ± SEM from 8 to 10 mice per group in **f**. ns, not significant, *p < 0.05, **p < 0.01, ***p < 0.001, unpaired Student’s *t-*test. Representative immunoblots in **a** and **c** are provided from three independent experiments. Unprocessed blots are in source data file

In further experiments, we confirmed that ZC3H18 plays a role in HR by showing that two independent siRNAs reduced ZC3H18 protein, disrupted DR-GFP recombination (Fig. 1a), and blocked the formation of RAD51 foci (Fig. 1b), a key event in HR repair, without disrupting the cell cycle (Supplementary Fig. 1c). Conversely, expression of an siRNA-resistant ZC3H18 rescued the HR defect in ZC3H18-depleted cells (Fig. 1c), indicating that the siRNA effect is due to ZC3H18 depletion. We also demonstrated that ZC3H18-depleted ovarian cancer cell lines (Supplementary Fig. 2a) were sensitive to the DNA crosslinkers cisplatin and melphalan as well as the PARP inhibitors olaparib and veliparib in culture (Fig. 1d, e; and Supplementary Fig. 2b). Consistent with the cell culture results, shRNA-mediated ZC3H18 depletion (Supplementary Fig. 2c) also sensitized xenografted OVCAR-8 cells to olaparib in mice treated with this PARPi (Fig. 1f). Collectively, these results demonstrate that *ZC3H18*, a gene located in a chromosomal region frequently deleted in HGSOC, is essential for HR and that ZC3H18 depletion sensitizes ovarian cancer cells to platinum agents and PARP inhibitors.

### ZC3H18 depletion reduces BRCA1, which drives the HR defect

**Fig. 2:**
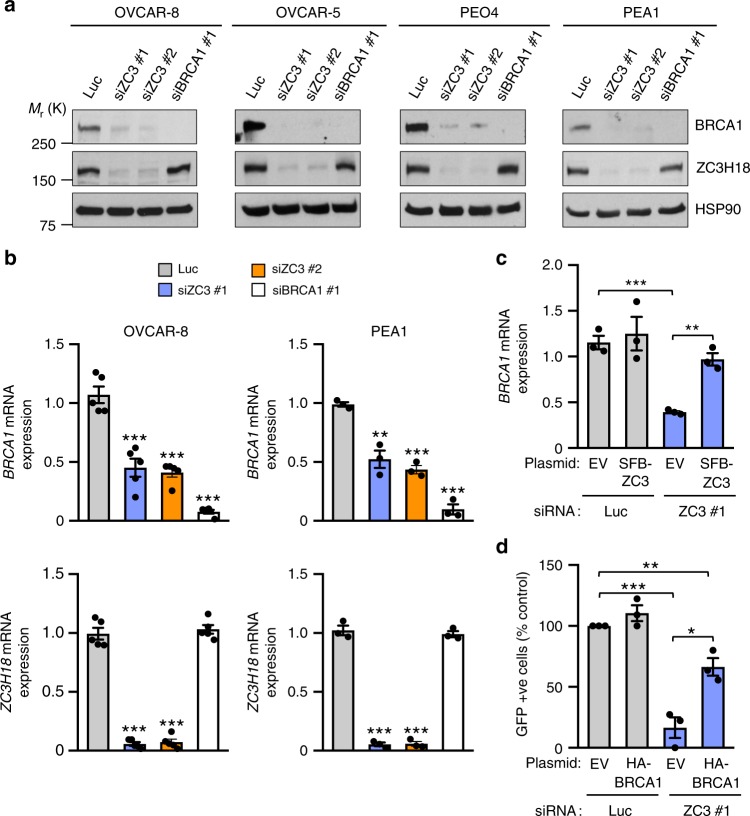
ZC3H18 depletion reduces *BRCA1* mRNA and protein levels. **a** and **b** Indicated cell lines were transfected with control luciferase (Luc), ZC3H18 (ZC3), or BRCA1 siRNAs. Forty-eight hours after transfection, the cells were immunoblotted for the indicated antigens (**a**) and analyzed by qRT-PCR for *ZC3H18* and *BRCA1* mRNA levels, which are expressed relative to *GAPDH* mRNA levels as an internal control (**b**). **c** Rescue of *BRCA1* mRNA and protein in ZC3H18-depleted OVCAR-8 cells with siRNA-resistant SFB-ZC3H18. OVCAR-8 cells were transfected with Luc or ZC3H18 siRNA plus empty vector (EV) or siRNA-resistant ZC3H18 plasmid (SFB-ZC3H18). *BRCA1* mRNA and proteins levels were assessed by qRT-PCR (**c**) and immunoblotting (Supplementary Fig. 4b), respectively. **d** Ectopic BRCA1 expression rescues the HR defect caused by ZC3H18 depletion. OCVAR-8 DR-GFP cells were transfected with empty vector (EV) or HA-BRCA1-expressing plasmid with either control luciferase (Luc) or ZC3H18 (ZC3) siRNAs. Seventy-two hours later, the cells were analyzed for GFP by flow cytometry. Representative immunoblots in **a** are provided from three independent experiments. Unprocessed blots are provided in Source data file. Shown are means ± SEM from three independent experiments in **b**–**d**. *p < 0.05, **p < 0.01, ***p < 0.001, unpaired Student’s *t-*test

Because ZC3H18 was previously shown to regulate gene expression through its effects on RNA metabolism^17,18,19,20^, we asked whether ZC3H18 depletion affected expression of genes associated with HR by RNA-seq (Supplementary Data 1). A KEGG pathway analysis^24^ showed that multiple HR-associated genes were downregulated (Supplementary Fig. 3), with BRCA1 among the most highly reduced by ZC3H18 depletion (Supplementary Data 1). Because defects in BRCA1 are the most frequent cause of HR deficiency and BRCA1 is a key regulator of HR^1^, we focused the present studies on BRCA1. Consistent with the RNA-seq analysis, ZC3H18 depletion profoundly decreased *BRCA1* mRNA (Fig. 2b; Supplementary Fig. 4a) and protein levels (Fig. 2a) in multiple ovarian cancer cell lines and in xenografted OVCAR-8 cells (Supplementary Fig. 2c). Moreover, expression of siRNA-resistant ZC3H18 restored *BRCA1* mRNA (Fig. 2c) and protein levels (Supplementary Fig. 4b) in ZC3H18 siRNA-transfected cells confirming that ZC3H18 facilitates accumulation of *BRCA1* mRNA and protein. Finally, because multiple HR-associated genes were downregulated by ZC3H18 depletion (Supplementary Data 1 and Supplementary Fig. 3), we next asked whether the loss of BRCA1 was a major contributor to the HR defect caused by ZC3H18 depletion. As shown in Fig. 2d, heterologous expression of HA-tagged BRCA1 (Supplementary Fig. 4c)  substantially restored HR, thus showing that the loss of BRCA1 is a major driver of the HR defect caused by ZC3H18 depletion.

### ZC3H18 depletion causes *BRCA1* promoter hypermethylation

Given that ZC3H18 was previously shown to affect RNA splicing and degradation^17–20^, we next assessed whether ZC3H18 regulates BRCA1 levels by altering these *BRCA1* mRNA processing events. In ZC3H18 siRNA-transfected ovarian cancer cells, we found no evidence of alternative *BRCA1* RNA splicing using a PCR-based method^25^ (Supplementary Fig. 5a). Additionally, *BRCA1* mRNA half-life was not affected by ZC3H18 depletion (Supplementary Fig. 5b). Similarly, depletion of ZCCHC8, which is a component of the ZC3H18 cap-binding complex that mediates RNA degradation^19^, did not alter *BRCA1* mRNA levels (Supplementary Fig. 5c). These results suggest that ZC3H18 mediates BRCA1 expression through a mechanism that differs from its previously identified functions.

**Fig. 3:**
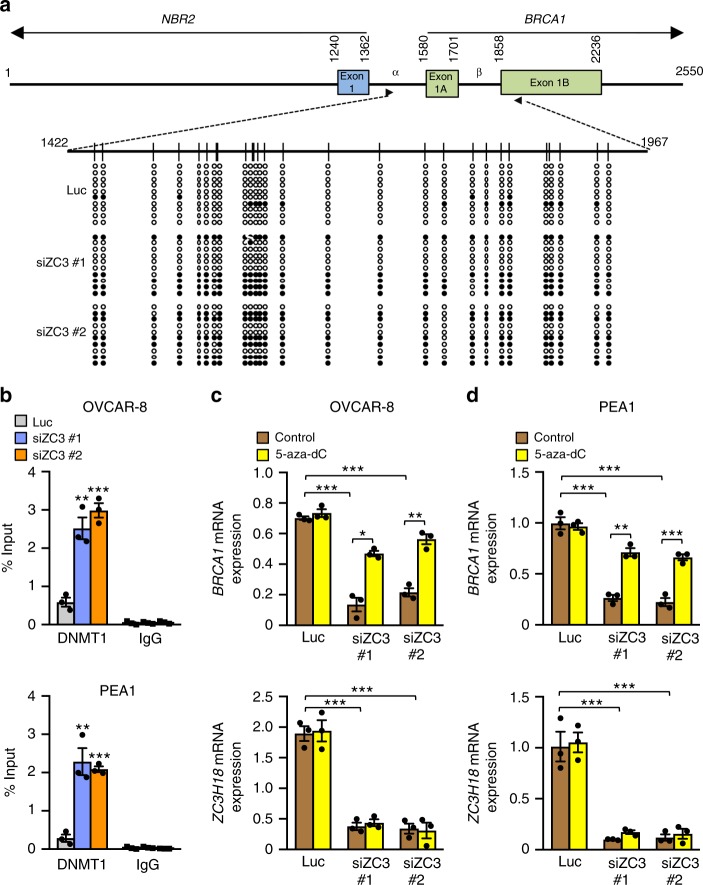
ZC3H18 depletion causes hypermethylation of the *BRCA1* promoter. **a** Map of the divergent *BRCA1/NBR2* promoter with CpG islands indicated by circles below the map. Methylation patterns obtained by bisulfite sequencing of 10 individual clones of PCR products from genomic DNA of control luciferase (Luc) and ZC3H18 siRNA (siZC3)-transfected OVACR-8 cells. Methylated (filled circles) and unmethylated (open circles) CpG positions are shown. **b** ChIP assays showing increased DNMT1 occupancy on the *BRCA1* promoter in ZC3H18-depleted cells. OVCAR-8 (top panel) and PEA1 (bottom panel) cells transfected with control luciferase (Luc) or ZC3H18 siRNAs were harvested 48 h after transfection and processed for ChIP to detect DNMT1 on the *BRCA1* promoter. **c** and **d** OVCAR-8 (**c**) and PEA1 (**d**) cells were transfected with control (Luc) or ZC3H18 siRNAs and treated with vehicle or 5-aza-2′-deoxycytidine (5 μM) for 3 days. *BRCA1* mRNA (top panel) and *ZC3H18* mRNA (bottom panel) levels were analyzed by qRT-PCR. The mRNA levels are normalized to *GAPDH* mRNA levels as the internal control. Data are means ± SEM from three independent experiments in **b**–**d**. *p < 0.05, **p < 0.01, ***p < 0.001, unpaired Student’s *t-*test

Because *BRCA1* is frequently silenced by promoter hypermethylation^6,8,26,27^, we next examined whether ZC3H18 depletion affected *BRCA1* promoter CpG methylation and the recruitment of DNA methyltransferase 1 (DNMT1), the prototypical DNA methyltransferase, to the *BRCA1* promoter. As previously reported^28^, bisulfite sequencing revealed that 10–20% of the *BRCA1* promoter CpG sites were methylated in control siRNA-transfected OVCAR-8 cells (Fig. 3a). In contrast, ZC3H18 depletion with two independent siRNAs increased *BRCA1* promoter methylation of these sites to ∼50% (Fig. 3a). Consistent with these results, chromatin immunoprecipitation (ChIP) assays showed that ZC3H18 depletion increased the accumulation of DNMT1 on the *BRCA1* promoter in two separate HGSOC cell lines, OVCAR-8 and PEA1 (Fig. 3b), without altering DNMT1 expression (Supplementary Fig. 5d). Conversely, treatment of the ZC3H18-depleted cells with the DNA methylation inhibitor 5-aza-2′-deoxycytidine (5-aza-dC) restored *BRCA1* mRNA levels without affecting *ZC3H18* mRNA levels (Fig. 3c, d), demonstrating that *BRCA1* promoter methylation induced by ZC3H18 depletion reduces *BRCA1* expression.

### ZC3H18 depletion promotes E2F1-mediated repression of *BRCA1*

**Fig. 4:**
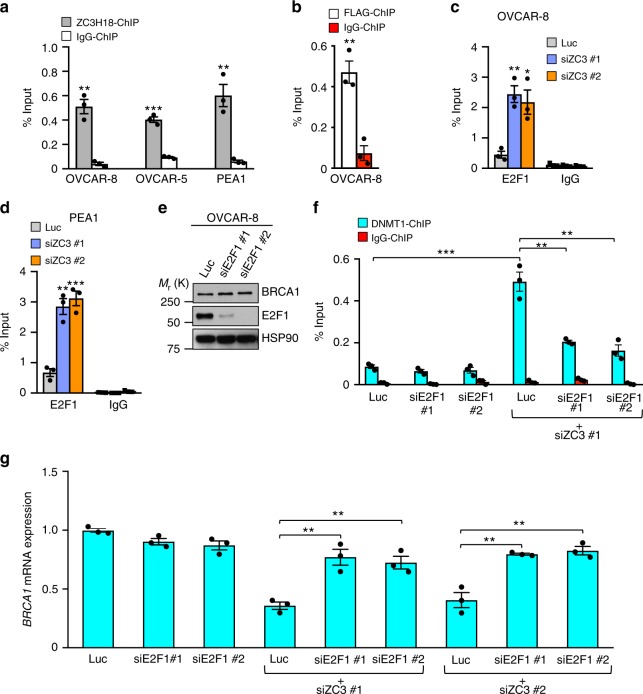
ZC3H18 occupies the *BRCA1* promoter and regulates E2F1. **a** OVCAR-8, OVCAR-5, and PEA1 cells were processed for ChIP assays using anti-ZC3H18 or IgG control antibodies and primers specific for the *BRCA1* promoter. **b** OVCAR-8 cells transiently expressing SFB-ZC3H18 were processed for ChIP assays using FLAG or IgG control antibodies. **c**, **d** ChIP assays showing increased occupancy of E2F1 on the *BRCA1* promoter when ZC3H18 is depleted. OVCAR-8 (**c**) and PEA1 (**d**) cells transfected with control luciferase (Luc) or ZC3H18 siRNAs (siZC3) were processed for ChIP using anti-E2F1 or IgG control antibodies 48 h after transfection. **e** E2F1 depletion does not affect BRCA1 levels. OVCAR-8 cells were transfected with control luciferase (Luc) or two independent E2F1 siRNAs and analyzed for BRCA1, E2F1, and HSP90 levels by immunoblotting 48 h later. **f** DNMT1 occupancy on the *BRCA1* promoter requires E2F1. OVCAR-8 cells transfected with Luc, E2F1, or ZC3H18 siRNA with our without E2F1 siRNAs were processed for ChIP with DNMT1 or control IgG antibodies 48 h after transfection. **g** Depletion of E2F1 restores BRCA1 expression when ZC3H18 is depleted. OVCAR-8 cells transfected with Luc, E2F1, or ZC3H18 siRNAs with our without E2F1 siRNAs were analyzed for *BRCA1* mRNA levels by qRT-PCR. *BRCA1* mRNA levels were normalized to *GAPDH* mRNA levels. Data in **a–g** are means ± SEM from three independent experiments. **p < 0.01, ***p < 0.001, unpaired Student’s *t-*test. Representative immunoblots in **e** are provided from three independent experiments. Unprocessed blots are provided in Source data file

To determine how ZC3H18 deficiency induces *BRCA1* promoter methylation, we first asked if ZC3H18 associates with the *BRCA1* promoter. ChIP of endogenous or overexpressed ZC3H18 demonstrated that ZC3H18 associates with the *BRCA1* promoter in multiple ovarian cancer cell lines (Fig. 4a, b), raising the possibility that ZC3H18 might affect the binding of transcription factors that regulate *BRCA1* expression.

E2F family members are key transcription factor regulators of the *BRCA1* gene that can either activate or repress transcription, depending on the family member that binds the promoter^29–31^. E2F1 was previously shown to activate *BRCA1* transcription in breast cancer and other cell types^30,32,33^. Accordingly, we asked whether ZC3H18 affects *BRCA1* expression by reducing E2F1 binding to the *BRCA1* promoter, an event predicted to reduce *BRCA1* expression. Suprisingly, however, ZC3H18 depletion greatly increased E2F1 binding to the BRCA1 promoter (Fig. 4c, d), suggesting that E2F1 was not activating *BRCA1* transcription in these cells. Consistent with this possibility, E2F1 depletion did not reduce *BRCA1* expression in ovarian cancer cell lines (Fig. 4e; Supplementary Fig. 6a) or in freshly isolated HGSOC tumors from two different mouse PDX models (Supplementary Fig. 6b, c) using primers that are specific for human *BRCA1* (Supplementary Fig. 6c), thus demonstrating that E2F1 does not play a major role in *BRCA1* transactivation in HGSOC.

Although E2F1 is generally considered to be a transcriptional activator, it can also repress transcription in some settings by recruiting the DNA methyltransferase DNMT1 to promoters^34^. Given that ZC3H18 depletion caused BRCA1 promoter methylation and E2F1 recruitment to the BRCA1 promoter, we reasoned that E2F1 might repress BRCA1 expression in ovarian cancer cells by recruiting DNMT1 when ZC3H18 was depleted. Consistent with this possibility, we found that E2F1 was required for DNMT1 recruitment to the *BRCA1* promoter (Fig. 4f). In additional experiments, E2F1 depletion restored BRCA1 expression in ZC3H18-depleted cells (Fig. 4g), further demonstrating that E2F1 mediates *BRCA1* repression in this setting.

These findings suggested that the role of E2F1 in *BRCA1* transcriptional regulation might differ between ovarian cancer cells, where E2F1 represses *BRCA1* transcription (Fig. 4f, g), and breast cancer cells, in which E2F1 promotes *BRCA1* transcription^30,32,33^. To further evaluate this possibility, we examined the effect of depleting E2F1 in MDA-MB-231 breast cancer cells. Consistent with previous reports in breast cancer cells, we found that E2F1 depletion reduced BRCA1 expression in MDA-MB-231 cells (Supplementary Fig. 7a). We next assessed the possibility that ZC3H18 might affect E2F1 and DNMT1 differently in ovarian and breast cancer cells. Indeed, ZC3H18 depletion enhanced E2F1 binding to DNMT1 in ovarian cancer but not in breast cancer cells (Supplementary Fig. 7b, c), suggesting that loss of ZC3H18 leads to E2F1-DNMT1 repressor complex formation in ovarian cancer cells. Despite the different roles of E2F1 in ovarian and breast cancer cells, ZC3H18 depletion reduced BRCA1 levels in both cell lines  (Supplementary Fig. 7a). Collectively, these results demonstrate that although ZC3H18 depletion reduces *BRCA1* expression in both ovarian and breast cancer cells, the mechanisms underlying ZC3H18 regulation of *BRCA1* differs in the two cells types, with ZC3H18 regulating the recruitment of E2F1 and DNMT1 to the *BRCA1* promoter to repress transcription in ovarian cancer cells.

### ZC3H18 facilitates E2F4 binding to the *BRCA1* promoter

**Fig. 5:**
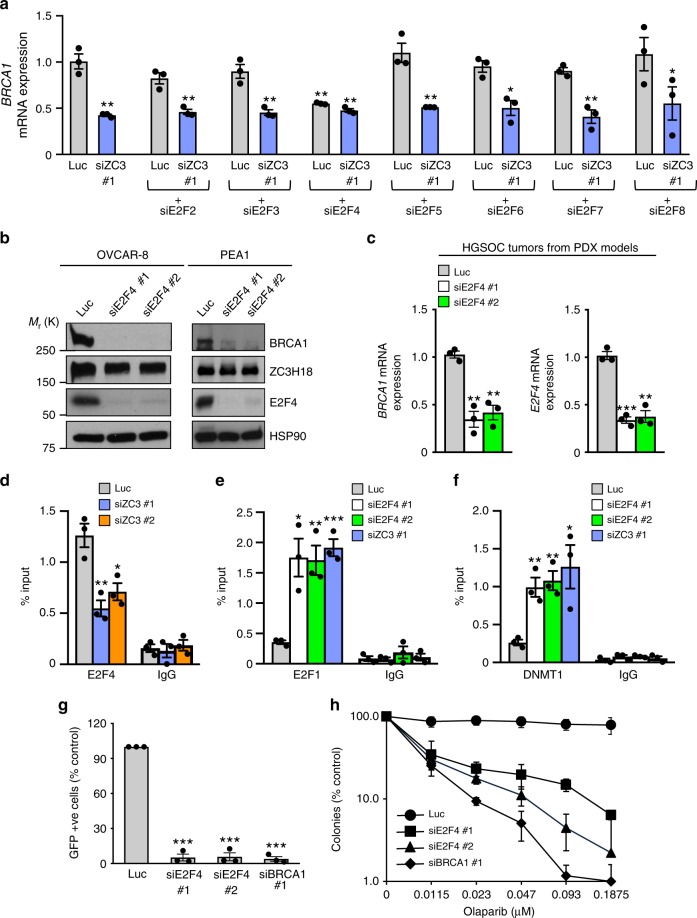
ZC3H18 promotes binding of E2F4 and activation of the *BRCA1* promoter. **a** qRT-PCR analysis of *BRCA1* mRNA expression. OVCAR-8 cells transfected with control luciferase (Luc), E2F2, E2F3, E2F4, E2F5, E2F6, E2F7, or E2F8 siRNAs with or without ZC3H18 siRNA were analyzed by qRT-PCR for *BRCA1* mRNA levels, which were normalized to *GAPDH* mRNA. **b** Immunoblots of indicated proteins in OVCAR-8 and PEA1 cells transfected with control luciferase (Luc) and two independent E2F4 siRNAs. **c** *BRCA1* mRNA expression, normalized to *GAPDH* mRNA, was determined by qRT-PCR in short-term ex vivo cultures of HGSOC tissues from PDX models electroporated with Luc or E2F4 siRNAs. **d** ZC3H18 promotes E2F4 occupancy on the *BRCA1* promoter. OVCAR-8 cells transfected with control luciferase (Luc) or ZC3H18 siRNAs were analyzed by ChIP for E2F4 bound to the *BRCA1* promoter. **e**, **f** Depletion of E2F4 or ZC3H18 promotes E2F1 and DNMT1 occupancy on the *BRCA1* promoter. OVCAR-8 cells transfected with Luc, E2F4, and ZC3H18 siRNAs were analyzed by ChIP for E2F1 (**e**) and DNMT1 (**f**) accumulation on the *BRCA1* promoter. **g** E2F4 depletion disrupts HR. OVCAR-8-DR-GFP cells transfected with pCβASceI plus indicated siRNAs were analyzed for GFP fluorescence by flow microfluorimitry 48 h after transfections. HR efficiencies were normalized to control (Luc) siRNA-transfected cells. **h** OVCAR-8 cells were transfected with control luciferase (Luc), E2F4, or BRCA1 siRNAs. Forty-eight hours later, the cells were trypsinized, re-plated, and allowed to adhere for 24 h. The indicated concentrations of olaparib were then added, and the cells were cultured for 10 days, stained with Coomassie Blue, and colonies were counted manually. Data are means ± SEM of three independent experiments. *p < 0.05, **p < 0.01, ***p < 0.001, unpaired Student’s *t-*test. Representative immunoblots in **b** are provided from three independent experiments. Unprocessed blots are provided in Source data file. The graph in **h** represents one of three independent experiments that gave similar results. Error bars are standard deviation of triplicate wells from a representative experiment

Because multiple E2F family members have been reported to regulate *BRCA1* transcription in a variety of cell line models^15,30,32,33^, we next explored the roles of the seven other known family members (E2F2-E2F8) in control and ZC3H18-depleted OVCAR-8 cells. These studies showed that (1) E2F4 was the only E2F family member that affected *BRCA1* expression and (2) co-depletion of E2F4 and ZC3H18 did not further suppress *BRCA1* mRNA levels (Fig. 5a), suggesting that E2F4 and ZC3H18 are in the same pathway. Additional studies showed that E2F4 depletion with two independent siRNAs reduced *BRCA1* mRNA and protein levels in OVCAR-8, PEA1, PEO1, and PEO4 cells without affecting ZC3H18 levels (Fig. 5b; Supplementary Fig. 8a, b). Similarly, E2F4 depletion also reduced BRCA1 expression in short-term ex vivo cultures of HGSOC tumors freshly isolated from three different PDX models (Fig. 5c).

Because the results in Fig. 5a and Supplementary Fig. 8a suggested that ZC3H18 and E2F4 regulate *BRCA1* through the same pathway, we next asked whether ZC3H18 and E2F4 affected one another’s interaction with the *BRCA1* promoter using ChIP. ZC3H18 depletion reduced E2F4 occupancy on the *BRCA1* promoter but did not affect E2F4 expression (Fig. 5d; Supplementary Fig. 8c, d). In contrast, E2F4 depletion did not alter ZC3H18 binding to the *BRCA1* promoter (Supplementary Fig. 8e), demonstrating that ZC3H18 enhances the recruitment of E2F4 to the *BRCA1* promoter but not vice versa. These findings also suggested that E2F4 might contribute to the effects of ZC3H18 on *BRCA1* expression. Consistent with this idea, E2F4 siRNAs increased E2F1 (Fig. 5e) and DNMT1 (Fig. 5f) recruitment to the *BRCA1* promoter, blocked HR (Fig. 5g), and sensitized cells to the PARP inhibitor olaparib (Fig. 5h) without disrupting the cell cycle (Supplementary Fig. 8f). Together, these results demonstrate that ZC3H18 enhances E2F4 binding to the *BRCA1* promoter, which concomitantly reduces binding of E2F1 and DNMT1 to the promoter and promotes *BRCA1* transcription.

### ZC3H18 and E2F4 bind adjacent E2F sites

**Fig. 6:**
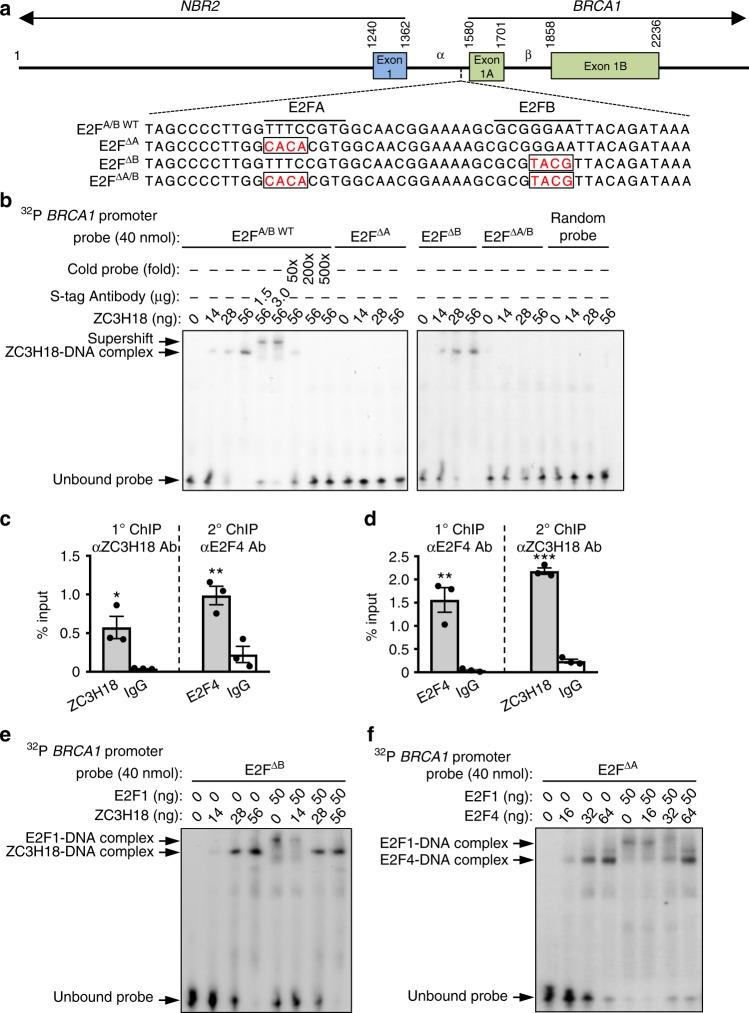
ZC3H18 binds the *BRCA1* promoter and inhibits E2F1 binding. **a** Schematic of the *BRCA1* proximal promoter with E2FA and E2FB sites indicated. Nucleotide sequences of the DNA probes used in the electrophoretic mobility shift assays (EMSA). E2FA and E2FB mutation sites are indicated in open rectangles. **b** EMSA with purified recombinant SFB-ZC3H18 using *BRCA1* promoter probe with wild-type sequence (E2FA/B^WT^) or mutations in the E2FA site (E2F^ΔA^), the E2FB site (E2F^ΔB^), or both E2F sites (E2F^ΔA/B^). A probe with randomly shuffled sequences was used as negative control. For supershift assays, an anti-S-Tag monoclonal antibody, which binds the SFB tag in SFB-ZC3H18, was used. **c**, **d** ZC3H18 and E2F4 co-occupy the endogenous *BRCA1* promoter. Sequential ChiP (ChIP-Re-ChIP) assays in OVCAR-8 cells using anti-ZC3H18 antibody for primary ChIP and anti-E2F4 antibody for secondary ChIP (**c**) and using anti-E2F4 antibody for primary ChIP and anti-ZC3H18 antibody for secondary ChIP (**d**). **e** EMSA with purified recombinant SFB-ZC3H18 and SFB-E2F1 using *BRCA1* promoter probe with mutated E2FB site (E2F^ΔB^). **f** EMSA with purified SFB-E2F4 and SFB-E2F1 using *BRCA1* promoter probe with mutated E2FA site (E2F^ΔA^). The images of EMSA in **b**, **e**, and **f** are representative of three independent experiments that gave similar results. Data in **c** and **d** are means ± SEM of three independent experiments. *p < 0.05, **p < 0.01, ***p < 0.001, unpaired Student’s *t-*test

Two key E2F binding sites, E2FA and E2FB^15,33^, have been identified in the bidirectional, ~250-bp region that drives transcription of *BRCA1* and the opposing gene *NBR2* (Fig. 6a). Mutation of either site disrupts *BRCA1* promoter activity in ovarian cancer cell lines (Supplementary Fig. 9a), demonstrating that both are required for full transcriptional activation of the *BRCA1* promoter in these cells. Based on the observation that ZC3H18 alters the binding of E2Fs to the *BRCA1* promoter, we next hypothesized that ZC3H18 directly binds to one of these E2F sites. To test this idea, we performed electrophoretic mobility shift assays (EMSAs) using bacterially expressed, purified ZC3H18 (Supplementary Fig. 9b). These studies showed that ZC3H18 directly binds a *BRCA1* promoter fragment with wild-type E2FA and E2FB sites (E2FA/B^WT^) (Fig. 6b). The specificity of the interaction was confirmed by cold probe competition, supershift assay, and random probe competition (Fig. 6b). Analyses using fragments with mutations in the E2FA (E2F^ΔA^), E2FB (E2F^ΔB^), or both (E2F^ΔA/B^) sites (Fig. 6a) showed that ZC3H18 binds the E2FA site but not the E2FB site (Fig. 6b). In contrast, purified E2F4 binds the E2FB site (Supplementary Fig. 9c, d), whereas purified E2F1 binds to both E2FA and E2FB sites (Supplementary Fig. 9e, f). In agreement with these in vitro observations, we found that E2F4 and ZC3H18 simultaneously occupy the *BRCA1* promoter in cells (Fig. 6c, d) using ChIP-Re-ChIP assays, which can detect the binding of two proteins on a single DNA sequence^35^.

We next investigated how ZC3H18 and E2F4 affect binding of E2F1, which represses *BRCA1* transcription, to the *BRCA1* promoter. We performed EMSAs using a fixed amount of E2F1 with increasing amounts of ZC3H18 or E2F4. Because ZC3H18 interacts with the E2FA site, we used the E2F^ΔB^ promoter probe to test the ability of ZC3H18 to regulate E2F1 binding. Similarly, because E2F4 interacts with the E2FB site, we used the E2F^ΔA^ promoter probe to test the ability of E2F4 to regulate E2F1 binding. Both ZC3H18 and E2F4 abolished E2F1 interaction with both E2F binding sites (Fig. 6e, f). These results suggest that ZC3H18 binding to the E2FA site — and E2F4 binding to the E2FB site — prevents the interaction of E2F1 with the *BRCA1* promoter, thereby blocking E2F1-mediated repression of *BRCA1*.

### *ZC3H18* and *E2F4* levels correlate with *BRCA1* levels in HGSOC

**Fig. 7:**
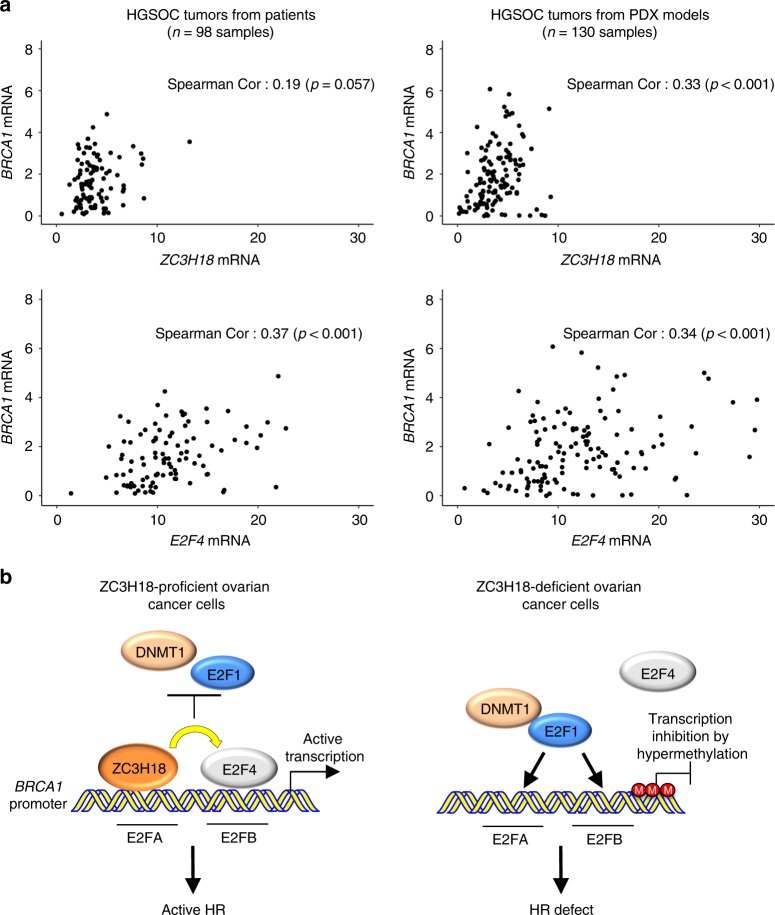
*ZC3H18* and *E2F4* expression correlates with *BRCA1* levels in HGSOC patient and PDX tumors. **a** Scatter plots of *BRCA1* mRNA expression as a function of either *ZC3H18* or *E2F4* mRNA expression in HGSOC tumors from patients and PDX models. mRNA expression is in RPKM units. **b** Model for the role of ZC3H18 in *BRCA1* transcription. Left panel: in ZC3H18-proficient cells, ZC3H18 directly binds to the E2FA site on the *BRCA1* promoter, where it promotes E2F4 occupancy at the E2FB site, thereby preventing E2F1-dependent DNMT1 occupancy and promoter methylation and inducing *BRCA1* transcription. Right panel: in ZC3H18-deficient cells, E2F1 occupies both E2FA and E2FB sites and causes DNMT1 loading onto the promoter, leading to methylation of the promoter, reduced expression of BRCA1, and disruption of HR. Spearman correlations are shown in the images

Our mechanistic studies discovered that ZC3H18 and E2F4 depletion reduces *BRCA1* levels in ovarian cancer cell lines and low-passage, short-term ex vivo-cultured HGSOC PDX models freshly isolated from mice (Figs. 2a, b, 5b, c). To further address whether ZC3H18 and E2F4 affect BRCA1 expression in HGSOC, we compared *BRCA1* mRNA levels with *ZC3H18* and *E2F4* mRNA levels in HGSOC tumors from patients and from PDX mouse models. This analysis showed that *BRCA1* mRNA levels were positively correlated with both *ZC3H18* and *E2F4* mRNA levels in patients (*ZC3H18*: *r* = 0.19, *p* = 0.057; *E2F4*: *r* = 0.37, *p* < 0.001) and PDX models (*ZC3H18*: *r* = 0.33, *p* < 0.001; *E2F4*: *r* = 0.34, *p* < 0.001) (Fig. 7a; Supplementary Data 2). Taken together, these findings suggest that ZC3H18 and E2F4 play a role in regulating BRCA1 expression in HGSOCs.

## Discussion

As summarized in Fig. 7b, we found that ZC3H18 is a DNA binding protein that regulates *BRCA1* transcription by directly interacting with the E2FA site in the *BRCA1* promoter. ZC3H18 binding to the E2FA site promotes E2F4 interaction with the adjacent E2FB. Co-occupancy of ZC3H18 and E2F4 on adjacent E2F sites prevents E2F1 binding to either E2F site and promotes *BRCA1* transcription. In contrast, when ZC3H18 is depleted, the E2FA and E2FB sites are instead occupied by E2F1, which represses *BRCA1* by recruiting DNMT1 and causing CpG hypermethylation at sites previously found to reduce BRCA1 expression in patients^6,27,36,37^. In agreement with these observations, loss of ZC3H18 reduces BRCA1 levels, disrupts HR, and sensitizes ovarian cancer cells to the DNA crosslinking agents, cisplatin and melphalan, as well as the PARP inhibitors veliparib and olaparib.

The E2F family of transcription factors consists of eight genes (*E2F1-8*) that encode nine different proteins^38^. E2F1, E2F2, and E2F3A can activate or repress transcription depending on whether they interact with pocket proteins, such as retinoblastoma (Rb), p107, and p130 that recruit DNMTs and other enzymes that silence target genes^32,34^. In contrast, E2F3B, E2F4, E2F5, E2F6, E2F7, and E2F8 were originally classified as repressors^38;^ however, additional studies have shown that E2F4 also activates transcription of multiple genes^29,39^. The roles of E2F family members in BRCA1 regulation have been primarily investigated in breast cancer cell lines^15,30,32,33^. Consistent with the typically accepted roles of E2F1 and E2F4, these studies have generally concluded that E2F1 activates *BRCA*1, whereas E2F4 represses *BRCA1* transcription. In contrast, our studies in multiple ovarian cancer cell lines found that E2F1 represses BRCA1 expression, and that this repression requires the DNA methyltransferase DNMT1 and is correlated with CpG hypermethylation of the *BRCA1* promoter.

We also found unexpected results with E2F4. Using an siRNA screen, we found that (1) of all the E2F family members, only E2F4 depletion reduced *BRCA1* expression in OVCAR-8 cells; (2) *BRCA1* expression in multiple ovarian cancer cell lines was reduced by two independent E2F4 siRNAs; (3) E2F4 depletion in short-term ex vivo cultures of freshly isolated HGSOC tumors from mouse PDX models reduced BRCA1 expression; and (4) *E2F4* expression is positively correlated with *BRCA1* expression in primary HGSOCs and ovarian cancer PDXs. Suprisingly, however, E2F4 was shown to repress *BRCA1* expression in breast cancer and other cell lines^32^. While we do not currently understand the underlying mechanism for this alternative regulation, E2F4 is converted into an activator when Rb family members are lost^40^. Accordingly, we speculate that differential expression of Rb family members and/or posttranslational modifications of Rb that regulate interactions with E2F family members may contribute to the disparate regulation of *BRCA1* in ovarian versus breast cancer. Taken together these results suggest that E2F4 is an activator of *BRCA1* transcription in ovarian cancer cells.

Notably, the present studies also uncovered a mechanism by which ZC3H18 regulates gene expression, namely that ZC3H18 is a DNA binding protein that interacts with a specific site in the *BRCA1* promoter. These findings add to the complex array of functions already ascribed to ZC3H18. These include activating the transcription factor NF-κB via an unknown mechanism^41^, regulating RNA metabolism by participating in mRNA splicing and export from the nucleus^18^, and targeting RNA for exosome-mediated degradation^19^. However, because (1) ZC3H18 depletion did not affect *BRCA1* mRNA splicing or stability (Supplementary Fig. 5a, b) and (2) ZCCHC8 depletion, which disrupts the CBCN complex, did not affect BRCA1 levels (Supplementary Fig. 5c), it is unlikely that ZC3H18 is regulating BRCA1 expression by altering the metabolism of BRCA1 RNAs. Instead, our results demonstrate that ZC3H18 directly binds DNA to activate *BRCA1* transcription. Consistent with our findings, while this manuscript was in preparation, Winczura et al.^17^ reported that ZC3H18 depletion reduced the transcription of a subset of genes, including *BRCA1*. Using ChIP assays, they also showed that ZC3H18 associates with the *BRCA1* promoter; however, the mechanism by which ZC3H18 increased *BRCA1* expression was not identified. Here we have identified a key mechanism by which ZC3H18 regulates the *BRCA1* promoter by showing that ZC3H18’s ability to bind directly to the *BRCA1* promoter and regulate the association of E2F family members is a major driver of *BRCA1* expression in ovarian cancer. However, as shown in Fig. 2a, b, ZC3H18 has a greater effect on BRCA1 protein levels than on its mRNA levels, suggesting that ZC3H18 may also regulate BRCA1 posttranscriptionally.

The findings presented here raise the possibility that *ZC3H18* loss contributes to HR defects by reducing BRCA1 expression. Consistent with this possibility, deep *ZC3H18* deletions and low *ZC3H18* mRNA levels are nearly mutually exclusive with *BRCA1* driver mutations and deep deletions in ovarian tumors analyzed by The Cancer Genome Atlas research network (Supplementary Fig. 10). This correlation was also observed in breast cancer (Supplementary Fig. 10), consistent with our observations that ZC3H18 regulates *BRCA1* in breast cancer cells. Accordingly, our findings suggest that loss and/or decreased ZC3H18 expression may help identify HGSOC patients most likely to benefit from PARP inhibitor and platinum-based therapies.

## Methods

### Cell lines, cell culture, and small molecules

The OVCAR-8 and OVCAR-5 cells were kind gifts from D. Scudierio (NCI, National Institutes of Health). OVCAR-8-DR-GFP cells, which have a genomically integrated DR-GFP substrate for HR repair assays, were described previously^42^. The PEA1 and PEO4 cells were from Sigma-Aldrich and Dr. T. Taniguchi (Fred Hutchinson Cancer Research Center), respectively. The cells were cultured in RPMI-1640 medium (Corning) supplemented with 8% fetal bovine serum (Millipore). All cells were authenticated by autosomal STR profiling (University of Arizona Genetics Core) and maintained in a humidified 37 °C incubator with 5% CO_2_. Cisplatin was obtained from Teva Pharmaceuticals. Melphalan, veliparib (ABT-888), and olaparib (AZD2281) were from Selleck Chemicals.

### siRNA transfections

siRNAs were purchased from Dharmacon. siRNA transfections (2 µM/transfection) were performed using a BTX ECM 830 electroporator. Cells (5 × 10^6^/transfection) were mixed with 20 µL of 20 µM siRNA solution in a 4-mm electroporation cuvette in 200 µL RPMI supplemented with 8% fetal bovine serum and electroporated with two 10-mS pulses at 280 V as previously described^42^. siRNAs used were:

luciferase, 5′-CUUACGCUGAGUACUUCGA-3′;

BRCA1, 5′-GUGGGUGUUGGACAGUGUA-3′;

E2F1 #1, 5′-UCGGAGAACUUUCAGAUCU-3′;

E2F1 #2, 5′-GAGAAGUCACGCUAUGAGA-3′;

E2F4 #1, 5′-GAGAUACCCUCUUGGCCAU-3′;

E2F4 #2, 5′-CAGAAGAAGUACCAGAUUC-3′;

ZC3H18 #1, 5′-GAAGCGCUAUGAACCAUCA-3′;

ZC3H18 #2, 5′-GAACGAGGACUCCGGCAUG-3′

siRNAs used in Supplementary Fig. S1A were siGENOME SMARTpool siRNAs (Dharmacon) that contain a pool of four different siRNAs that target various regions of each gene.

### Plasmids and transfections

Human *ZC3H18* cDNA (Dharmacon, MHS6278-202759301), *E2F4* cDNA (Addgene plasmid #10914)^43^, and *E2F1* cDNA (Addgene plasmid #24225)^44^ were subcloned into the pSFB vector that contains in-frame N-terminal S-peptide, FLAG, and streptavidin-binding peptide tags^45^. Luciferase reporter assays used pBRC-FF, which contains the *BRCA1* promoter driving firefly luciferase expression (a kind gift from Dr. Peter Glazer, Yale University)^15^. To introduce E2FA and/or E2FB site mutations into pBRC-FF, PCR-based site-directed mutagenesis was performed using the following: for E2FA site mutation, 5′-CGGTAGCCCCTTGGCACACGTGGCAACGGAAAAG-3′ (sense) and 5′-CTTTTCCGTTGCCACGTGTGCCAAGGGGCTACCG-3′ (antisense); for E2FB site mutation, 5′-CCGTGGCAACGGAAAAGCGCGTACGTTACAGATAAATTAAAACTG-3′ (sense) and 5′-CAGTTTTAATTTATCTGTAACGTACGCGCTTTTCCGTTGCCACGG-3′ (antisense). The pRL-SV40 *Renilla* luciferase reporter construct was from Promega. Transfection of plasmids was as described^42^. For *E. coli* expression and purification of human ZC3H18, N-terminally SFB-tagged ZC3H18 was subcloned into the pET-24a(+) expression vector with an in-frame C-terminal 6X His-Tag (Novagen). An HA-tagged human full-length *BRCA1* plasmid construct was described before^46^. The pCßASceI plasmid was obtained from Addgene (Addgene plasmid #26477)^47^ . All plasmid constructs generated were confirmed by Sanger sequencing.

Plasmid transfections were performed as described for siRNA transfections above except that transfections used 1–1.5 × 10^7^ cells and 40 µg of plasmid (using a combination of empty vector and gene of interest to obtain 40 µg) per transfection.

### MTS and clonogenic assays

Twenty-four hours after siRNA transfection, cells were seeded in 96-well plates (1.2 × 10^5^ cells/well) and incubated for additional 24 h. The cells were then treated with the DNA crosslinking agents, cisplatin, or melphalan, for 3 days or PARP inhibitors, veliparib or olaparib, for 7 days. Cell viability following drug exposure was detected using MTS [(3-(4,5-dimethylthiazol-2-yl)-5-(3-carboxymethoxyphenyl)-2-(4-sulfophenyl)-2H-tetrazolium)/phenazine methosulfate (PMS)] colorimetric assay (Promega) according to the supplier’s protocol. For clonogenic assays, 48 h after siRNA transfection, OVCAR-8 cells were seeded in 6-well plates at 300 cells/well (in triplicate per assay point) and allowed to adhere overnight. The cells were then treated with the indicated concentrations of olaparib, and cultured in the presence of the drug for 8–10 days. Colonies were stained with Coomassie Blue, and colonies of >50 cells were counted manually. Inhibition of colony formation was presented as percentage of colonies formed compared to corresponding untreated control.

### Cell cycle analysis

Forty-eight hours after siRNA transfection, cells were harvested, fixed with ethanol, and cell cycle analysis was conducted by flow cytometry following staining of the DNA with propidium iodide.

### Immunocytochemistry

Forty-eight hours after siRNA transfection, cells were harvested and plated onto 8-well chamber slides (ThermoFisher Scientific), and the cells were allowed to attach for another 24 h. The cells were then irradiated with 2 Gray of ionizing radiation, incubated at 37 °C for 6 h, fixed with 3% paraformaldehyde in phosphate buffered saline (PBS) for 12 min, and permeabilized in 0.25% Triton X-100 in PBS for 10 min. The slides were blocked with 3% bovine serum albumin in PBS containing 0.25% Triton X-100, incubated at room temperature overnight with rabbit polyclonal primary antibody to RAD51 (1:250, PC-130, Calbiochem) and mouse monoclonal antibody to phospho-histone H2A.X (γ-H2A.X) (1:250, 05-636, Millipore), washed three times with PBS, incubated with anti-rabbit Alexa Fluor® 488 and anti-mouse Alexa Fluor® 594–conjugated secondary antibodies (1:500; ThermoFisher Scientific) for 1 h in the dark, washed once with PBS, incubated with Hoechst-33342 (1:1000, ThermoFisher Scientific) for 2 min to stain the nuclei, mounted in Prolong Gold Antifade (ThermoFisher Scientific), and examined with a confocal laser scanning microscope using a ×40 or ×100 objective.

### Immunoblotting

Two days after siRNA transfection, cells were harvested and lysed in 50 mM HEPES (pH 7.6), 150 mM NaCl, 1 mM EDTA, 1% Triton X-100, 10 mM NaF, 30 mM sodium pyrophosphate, 1 mM Na3VO4, 10 mM 2-glycerophosphate, 10 μg/mL leupeptin, 5 μg/mL aprotinin, 5 μg/mL pepstatin, and 20 mM microcystin-LR. Immunoblotting was done using the following primary antibodies: rabbit polyclonal ZC3H18 (1:1000, A304-682A, Bethyl Laboratories Inc.); mouse monoclonal BRCA1 (1:2000, sc-6954, Santa Cruz Biotechnology); mouse monoclonal E2F1 (1:500, ab4070, Abcam); rabbit polyclonal E2F4 (1:1000, NBP1-21374, Novus Biologicals); mouse monoclonal DNMT1 (1:5000, ab13537, Abcam); mouse monoclonal HSP90 (D. Toft, Mayo Clinic, H9010), and rabbit monoclonal HA-tag (1:1000, CST-3724S, Cell Signaling Technology). Secondary antibodies used were: horseradish peroxidase-conjugated anti-mouse immunoglobulin G (1:2000 for BRCA1 and 1:10,000 for all other primary antibodies, 7076 S, Cell Signaling Technology) and anti-rabbit immunoglobulin G (1:16,000 for ZC3H18 and 1:10,000 for all other primary antibodies, 7074 S, Cell Signaling Technology).

### HR assays

HR assays were performed using OVCAR-8-DR-GFP cells^42^. The cells were transfected twice. On day 1, they were transfected with siRNAs only. On day 2, they were transfected with the same siRNAs as on day 1 along with 20 μg pCßASceI plasmid (encoding I-SceI) with empty vector (pcDNA3) or expression vectors for SFB-ZC3H18 or HA-BRCA1. GFP fluorescence was assessed by flow cytometry on day 5.

### RNA extraction, cDNA synthesis, and quantitative real-time PCR (qPCR)

Total RNA was extracted from frozen cells using the miRNeasy mini kit (Qiagen) following the supplier’s instructions. The extracted RNA was converted to cDNA using oligo(dT) primers and SuperScript™ III reverse transcriptase (ThermoFisher Scientific). Quantitative PCR was performed in triplicate for each sample on a CFX96 real-time PCR system (Bio-Rad) using iTaq Universal SYBR Green Supermix (Bio-Rad). Expression was normalized to GAPDH. The qPCR primers used were:

*BRCA1:*

Forward: 5′-GCCAAGGCAAGATCTAGAGG-3′

Reverse: 5′-GTTGCCAACACGAGCTGA-3′

*ZC3H18:*

Forward: 5′-TCCCGGTCTTCATCCTACAG-3′

Reverse: 5′-CCGGCTTCTTCACTGACTTC-3′

*E2F4*:

Forward: 5′-CATAGGGGGCAGTGTCTTGT-3′

Reverse: 5′-CTAAAGGCCCAGCAGAAGTG-3′

*ZCCHC8*:

Forward: 5′-CTGGCCGGTGCATATAACTT-3′

Reverse: 5′-TGTACCACCACACCTGGCTA-3′

*GAPDH:*

Forward: 5′-GAAGGTGAAGGTCGGAGTCA-3′

Reverse: 5′-AATGAAGGGGTCATTGATGG-3′

### Bisulfite sequencing

Cells were harvested 48 h after siRNA transfection, and genomic DNA was extracted using phenol-chloroform. Genomic DNA (2 µg) was modified with bisulfite using the EpiMark® Bisulfite Conversion Kit (New England Biolabs) according to the supplier’s protocol. Bisulfite-modified DNA (40 ng) was amplified by PCR using Platinum™ *Taq* DNA polymerase and primer pairs (see below) to cover the *BRCA1* promoter (GenBank Accession No. U37574). The PCR products were run on a 1% agarose gel, excised, extracted using QIAquick Gel Extraction Kit (Qiagen), and subcloned into pCR™ 2.1-TOPO® TA vector using TOPO® TA Cloning® Kit (ThermoFisher Scientific). At least 10 clones of each PCR product were subjected to Sanger sequencing, and CpG methylation was analyzed by Quantification Tool for Methylation Analysis (QUMA)^48^. PCR primers specific for bisulfite-converted *BRCA1* promoter were designed using MethPrimer software^49^. The positions of the primers are shown in Fig. 3a.

Promoter region 1422–1967:

Forward: 5′-AGATTGGGTGGTTAATTTAGAGTTT-3′

Reverse: 5′-ATAATATCCCCCTCAAAACATATTC-3′

The reverse primer was used for Sanger sequencing.

### Chromatin immunoprecipitation (ChIP) assays

Cells (1 × 10^7^) in 15-cm dishes were cross-linked with 1% formaldehyde in media for 10 min at room temperature, and the unreacted formaldehyde was quenched by the addition of one-tenth volume of 1.25 M glycine (pH 7.0). The cells were washed with PBS, dislodged by scraping, collected by centrifugation at 800 × *g* for 5 min at 4 °C, resuspended in cell lysis buffer (10 mM Tris HCl, pH 7.5, 10 mM NaCl, 0.5% NP-40), and incubated on ice for 15 min. The pellet (chromatin) was digested with micrococcal nuclease (2.5 units/ml; New England Biolabs) for 20 min at 37 °C and sonicated for 15 min using the Diagenode Bioruptor-300 sonication system. Aliquots of sheared chromatin were immunoprecipitated using protein G Dynabeads™ and 1 μg of the above-mentioned ZC3H18, DNMT1, E2F1, or E2F4 antibodies. Immunoprecipitation with normal mouse IgG (1 μg/ChIP, 0107-01, SouthernBiotech) or rabbit IgG (1 μg/ChIP, 0111-01, SouthernBiotech) were used as negative controls. After immunoprecipitation, crosslinks were reversed by heating to 60 °C, and immunoprecipitated DNA was purified using spin columns (Cat. No. 11732676001, Roche). qPCR analysis of the ChIP and genomic input DNAs was performed using iQ™ SYBR® Green Supermix (Bio-Rad) using the supplier’s protocol. The following primers that amplify the *BRCA1* promoter region that contains the E2FA and E2FB sites were used: forward, 5′-CTTGATTTCGTATTCTGAGAGG-3′ and reverse, 5′-GCTGTGGGGTTTCTCAGATA-3′. For the ChIP-Re-ChIP assay, ChIP assays were first performed as described above, except that each ChIP was done in duplicate and eluates from the duplicate ChIPs were pooled to enhance the signals obtained in the second ChIP. Forty microliters (1/5th volume) from the first ChIP was used for subsequent analysis of the first immunoprecipitation. The remaining solution was diluted in 500 μL of IP dilution buffer (0.01% SDS, 1.1% Triton X-100, 16.7 mM Tris-HCl at pH 8.1, 167 mM NaCl) and immunoprecipitation was performed using the second antibody, and the immunoprecipitated DNA was subsequently analyzed by qPCR as described above.

### Protein purification

To produce recombinant full-length ZC3H18 protein, *E. coli* strain BL21(DE3) transformed with the SFB-ZC3H18-pET24a(+) construct was cultured in 250 ml LB medium at 37 °C until it reached an OD600 between 0.5 and 0.8. The culture was transferred to a 16 °C shaking incubator, 0.5 mM isopropyl-β-d-thiogalactopyranoside (IPTG) was added, and the culture was incubated overnight. The cells were harvested by centrifugation, resuspended in PB (50 mM sodium phosphate, pH 7.4, 300 mM NaCl) containing 1 mM imidazole and sonicated on ice. The lysates were cleared by centrifugation at 14,000 × *g* for 30 min at 4 °C and incubated with Ni-NTA His-Bind Superflow beads (Novagen) for 1 h at 4 °C. The beads were washed 3 times with PB containing 1 mM imadazole and eluted with PB containing 150 mM imidazole (pH 7.4) at 4 °C. The eluate was then exchanged into NETN buffer (20 mM Tris-HCl, pH 8.0, 100 mM NaCl, 0.5% NP-40, and 1 mM EDTA) using an Amicon® Ultra-15 centrifugal filter unit system (Sigma) and incubated with anti-FLAG M2 antibody (F3165, Sigma) and protein G (ThermoFisher Scientific) for 2 h at 4 °C. The beads were washed five times with NETN buffer (20 mM Tris-HCl, pH 8.0, 100 mM NaCl, 0.5% NP-40, and 1 mM EDTA), and eluted with 20 μg 3xFLAG peptide (Sigma) by centrifugation at 2000 × *g* for 3 min at 4 °C.

To produce E2F4 and E2F1 proteins, pSFB-E2F4 or pSFB-E2F1 plasmids (40 μg/transfection) were electroporated into K562 cells. Cells were harvested 24 h after transfection, and lysed with RIPA buffer (150 mM NaCl, 1% NP-40, 0.5% sodium deoxycholate, 0.1% SDS, and 50 mM Tris, pH 8.0). The clarified lysate was incubated with anti-FLAG M2 antibody and protein G agarose affinity resin (ThermoFisher Scientific) for 4 h, washed 5 times in RIPA buffer, and SFB-tagged proteins were eluted with 20 µg 3xFLAG peptide in elution buffer (50 mM Tris, pH7.4, 5 mM MgCl_2_, 150 mM NaCl). The purity of the eluted proteins was determined by Coomassie Blue (Bio-Rad) or SYPRO® Ruby staining (ThermoFisher Scientific) in accordance with supplier’s instructions.

### Electrophoretic mobility gel-shift assay

Complementary 50-bp single-stranded oligonucleotides corresponding to the *BRCA1* promoter with wild-type E2FA and E2FB sites or mutations in E2FA, E2FB, or both E2FA and E2FB sites were synthesized by Integrated DNA Technologies. The complementary oligonucleotides were end-labeled with γ-^32^P-ATP using polynucleotide kinase (NEB), and purified using CHROMA SPIN^TM^ + TE-10 columns (Takara). Equimolar concentrations of each oligonucleotide were diluted into annealing buffer (20 mM Tris-HCl, pH 8.0, 50 mM NaCl, 1 mM MgCl_2_) in a microcentrifuge tube, heated to 100 °C in a water bath, and cooled slowly to 4 °C to anneal the oligonucleotides. EMSA assays were performed by incubating the duplexed oligonucleotides in gel-shift buffer (20 mM Tris-HCl, pH 8.0, 50 mM NaCl, 1 mM MgCl_2,_ 5 mg/ml BSA, and 1 mM dithiothreitol) and various concentrations of purified ZC3H18, E2F1, and/or E2F4 proteins for 20 min at room temperature. For cold competition, the binding reaction mixture was pre-incubated with 50-, 200-, or 500-fold excess unlabeled probe for 10 min before adding the labeled probe. For supershift assays, the reaction mixture was pre-incubated with anti-S-tag antibody^50^, which recognizes the N-terminal S-peptide of the SFB-tagged proteins, for 30 min on ice before adding the labeled DNA probe. Samples were run on a 5% native acrylamide gel in 0.25 × TBE (0.0225 M Tris-borate, 0.0005 M EDTA pH 8.0) at 4 °C. The gel was dried, and autoradiography was performed.

### BRCA1 splicing analysis

Fourty-eight hour after siRNA transfection, total RNA was extracted and cDNA was synthesized as described above. The cDNA was amplified by PCR using Platinum™ *Taq* DNA polymerase and primer pairs^25^ to cover all the *BRCA1* exons.

The primers used were: Exon 1A–3: forward, 5′-GACAGGCTGTGGGGTTTCT-3′;

reverse, 5′-TTTGTGGAGACAGGTTCCTTGA-3′

Exon 1A–6: forward, 5′-GACAGGCTGTGGGGTTTCT-3′;

reverse, 5′-TCCAAACCTGTGTCAAGCTG-3′

Exon 1A–11q: forward, 5′-GACAGGCTGTGGGGTTTCT-3′;

reverse, 5′-TGG CTCCACATGCAAGTTTG-3′

Exon 7-11q: forward, 5′-CATCCAAAGTATGGGCTACAG-3′;

reverse, 5′-TGG CTCCACATGCAAGTTTG-3′

Exon 7-12: forward, 5′-CATCCAAAGTATGGGCTACAG-3′;

reverse, 5′-CTGAGAGGATAGCCCTGA-3′

Exon 8-13: forward, 5′-GGTTGTATCCGCTGCTTTGT-3′;

reverse, 5′- ATGGAAGGGTAGCTGTTAGAAGG-3′

Exon 10–12: forward, 5′-CCAGGGATGAAATCAGTTTGG-3′;

reverse, 5′-GCGTCTCTGAAGACTGCTCA-3′

Exon 10–13: forward, 5′-CCAGGGATGAAATCAGTTTGG-3′;

reverse, 5′-ATGGAAGGGTAGCTGTTAGAAGG-3′

Exon 12-13: forward, 5′-GCGTCTCTGAAGACTGCTCA-3′;

reverse, 5′-ATGGGAGCCAGCCTTCTAAC-3′

Exon 12-14: forward, 5′-GCGTCTCTGAAGACTGCTCA-3′;

reverse, 5′-AAAGGCCTTCTGGATTCTGG-3′

Exon 12-16: forward, 5′-GCGTCTCTGAAGACTGCTCA-3′;

reverse, 5′-CTCACACTTTCTTCCATTGC-3′

Exon 13-22: forward, 5′-ATGGGAGCCAGCCTTCTAAC-3′;

reverse, 5′-CACAGCTGTACCATCCATTC-3′

Exon 14-22: forward, 5′-TCTGCAGATAGTTCTACCAG-3′;

reverse, 5′-CACAGCTGTACCATCCATTC-3′

Exon 16-22: forward, 5′-AAAGAATGTCCATGGTGGTG-3′;

reverse, 5′-CACAGCTGTACCATCCATTC-3′

Exon 16-24: forward, 5′-AAAGAATGTCCATGGTGGTG-3′;

reverse, 5′- ACCACAGGTGCCTCACACAT-3′

Exon 20-24: forward, 5′- AGAAACCACCAAGGTCCAAAG-3′;

reverse, 5′- ACCACAGGTGCCTCACACAT-3′

The PCR products were run on a 1% agarose gel to visualize the alternative *BRCA1* splicing pattern.

### Luciferase reporter assay

OVCAR-8 cells were transfected with *BRCA1*-promoter firefly luciferase constructs (2 μg/transfection) and an internal control for transfection efficiency (pRL-SV40 *Renilla* luciferase reporter construct, Promega, 100 ng/transfection) and plated into 6-well plates. Samples were harvested 24 h after transfection, and luciferase activity was measured using Dual-Glo® luciferase assay system (Promega) following the supplier’s protocol. To control for intersample variations in transfection efficiencies, firefly luciferase readouts were normalized to *renilla* luciferase readouts.

### Ex vivo culture of HGSOC tumor tissues from PDX mouse models

To obtain short-term, 2D, ex vivo monolayer cultures of tumor cells, HGSOC tissues from PDX mouse models were harvested, minced into 2–4-mm pieces with a sterile scalpel blade, and dissociated using a tumor dissociation kit (Cat. # 130-096-730, Miltenyi Biotec) following the supplier’s protocol. After dissociation, the cells were washed five times with RPMI-1640 medium (Invitrogen) supplemented with 10% fetal bovine serum (Invitrogen), 100 units/mL penicillin and 100 units/mL streptomycin (Invitrogen), resuspended in RPMI-1640 medium with 10% fetal bovine serum without antibiotics, and electroporated with control luciferase (Luc), E2F1, or E2F4 siRNAs as described^42^. The cells were then plated in 24-well plates in RPMI-1640 supplemented with 10% fetal bovine serum and 100 units/mL penicillin and 100 units/mL streptomycin (Invitrogen). The plates were cultured for 48 h and harvested for RNA extraction and qRT-PCR.

### Analyses of patient and PDX tumors

Fresh tissues from high-grade serous ovarian, primary peritoneal, or fallopian tube cancers were collected at the time of primary debulking surgery at Mayo Clinic, Rochester from chemotherapy naïve patients who provided written and informed consent. All biospecimens were coded with a patient heterotransplant (PH) number to protect patient identity in accordance with the Mayo Clinic Institutional Review Board and in accordance with the Health Insurance Portability and Accountability Act regulations through the Mayo Clinic Ovarian Tumor Repository. PDX models were developed as previously described by intraperitoneal injection into female SCID beige mice (C.B-17/IcrHsd-*Prkdc*^*scid*^ *Lyst*^*bg-J*^; ENVIGO), also in accordance with the Mayo Clinic Institutional Animal Care and Use Committee. Briefly, 0.1 to 0.3 cc of minced fresh patient tumor was mixed 1:1 with McCoy’s media with rituximab^51^ in a 1-mL syringe and injected intraperitoneally through a 0.5-inch 16-gauge needle. No enzymatic or mechanical tumor dissociation was performed. Mice were monitored by routine palpation for engraftment and when moribund, tumors were snap frozen for subsequent studies. For primary patient samples, surplus tumor tissue in excess of requirements to generate PDXs was also snap frozen for future RNA work.

Total RNA was isolated from tissues collected from 97 patients and 138 non-overlapping PDX from mice using the RNeasy Micro kit (Qiagen, #74004) according to the manufacturer instructions. Purification of total RNA concentration and purity was determined on a Thermo Scientific NanoDrop 2000c UV-Vis Spectrophotometer (Thermo Scientific, Wilmington, DE). All samples met RNA integrity number and validated Agilent (Agilent Technologies, Santa Clara, CA) criteria.

RNA libraries were prepared according to the manufacturer’s instructions for the TruSeq RNA Sample Prep Kit (Illumina, San Diego, CA, USA). The concentration and size distribution of the libraries were determined on an Agilent Bioanalyzer DNA 1000 chip (Santa Clara, CA, USA). Libraries were loaded onto flow cells at concentrations of 8–10 pM to generate cluster densities of 700,000/mm^2^ following Illumina’s standard protocol using the Illumina cBot and cBot Paired End cluster kit version 3. The flow cells were sequenced as 51 × 2 Paired End reads on an Illumina HiSeq 2000 using TruSeq SBS sequencing kit version 3 and SCS version 1.4.8 data collection software. Base calling was performed using Illumina’s RTA version 1.12.4.2. There were ~45 million reads per sample mapped to the human genome, and 21,686 genes were detected. mRNA levels are expressed as RPKM (reads per kilobase per million mapped reads) using the formula: (10^9^×count)/(total reads×feature length), where count is the number of reads mapping to the gene or exon, total reads is the total number of reads mapping to all genes or exons in that sample, and feature length is the length of the gene or exon. Spearman correlation was used to assess correlation between *BRCA1* mRNA levels with *ZC3H18* and *E2F4* mRNA.

### RNA-seq analyses of OVCAR-8 cells

Forty-eight hour after control luciferase, ZC3H18 #1, or ZC3H18 #2 siRNA transfection, RNA was isolated using miRNeasy mini kit (Qiagen). Three independent RNA samples were prepared for each of the transfected siRNAs. Libraries were prepared (Illumina TruSeq mRNA v2) and processed through Mayo Clinic’s MAP-RSeq (v2.1.0) application^52^. The gene counts were generated by FeatureCounts^53^ using Ensembl’s hg19 gene definition file. RSeqQC^54^ was used to create quality control metrics, including gene body coverage plots, to insure the results from each sample were reliable and could be collectively used for a differential expression analysis. Genes with an average of <25 reads were removed from the differential expression analysis. The R package (v3.3.1), edgeR^55^ was used to identify which genes were differentially expressed. Statistically significant genes were defined by having a false discovery rate below 0.5. These differentially expressed genes were then used in a hypergeometric gene set enrichment test to evaluate enrichment in publically defined homologous recombination genes. The publically defined homologous recombination genes were defined by KEGG^24^ and the Gene Ontology^56^.

### In vivo PARPi efficacy studies

OVCAR-8 cells with doxycycline-inducible control non-targeting or ZC3H18 shRNAs were produced by lentiviral transduction. OVCAR-8 cells were seeded at 5 × 10^4^ cells/well in six-well plates with 2 mL of complete medium (RPMI-1640 supplemented with 8% FBS) and cultured overnight at 37 °C. The culture medium was replaced with 1:1 mixture of complete medium containing 2 μg/mL polybrene and TRIPZ doxycycline-inducible non-targeting shRNA control (shNT; Cat. No. RHS4743, Dharmacon) or ZC3H18 shRNA (shZC3, Cat. No. V2THS_19374, Dharmacon) lentiviral particle stocks. After 48 h, the medium was replaced with complete medium containing 0.75 µg/mL puromycin to select for stable shNT and shZC3 genomically integrated populations.

All animal studies and procedures were reviewed and approved by the Mayo Clinic Institutional Animal Care and Use Committee (IACUC). Immunocompromised 8-week-old SCID Beige female mice (C.B-17/IcrHsd-*Prkdc*^*scid*^ *Lyst*^*bg-J*^; ENVIGO) were inoculated with 5 × 10^6^ stable shNT or shZC3 OVCAR-8 cells intraperitonially in 200 µL serum-free RPMI-1640 medium. After 5 days, mice were given doxycycline chow (ENVIGO), which was continued until the end of the xenograft study. After 3 days, mice inoculated with shNT- or shZC3-transduced cells were assigned randomly into two groups (10 mice per group) that received vehicle control (0.5% methyl cellulose) or 50 mg/kg olaparib daily by oral gavage. After 28 days of treatment, tumors were weighed, snap frozen in liquid N_2_, and ground with mortar and pestal. In total, 10 mg of tumor tissue was then lysed in SDS-PAGE sample buffer, and protein concentrations were determined by Coomassie Blue staining of pilot SDS-PAGE gels. Equal protein concentrations from each tumor were then immunoblotted for ZC3H18, BRCA1, and HSP90.

### 16q24.2 copy number and *ZC3H18* and *BRCA*1 alterations in HGSOC

Analysis of 16q24.2 copy number changes in HGSOC was performed using the GDC TCGA Ovarian Cancer dataset and the UCSC Xena Browser (https://xenabrowser.net)^57^.

Potential mutual exclusivity analysis of *BRCA1* deletions and driver mutations versus *ZC3H18* deletions and low *ZC3H18* mRNA levels was performed using genomics data in the cBioPortal (https://www.cbioportal.org)^58,59^. *ZC3H18* deep (homologous) deletions and mRNA expression <2 standard deviations below mean versus deep (homologous) deletions and driver mutations for *BRCA1* in ovarian serous cystadenocarcinoma and breast invasive carcinoma were compared using TCGA PanCancer Atlas databases. OncoPrints of the alterations generated in the cBioPortal are presented.

### Statistics

Data in bar and line graphs are reported as means ± SEM. Statistical analyses were performed in GraphPad Prism Version 8.0 (GraphPad Software, La Jolla, CA, USA) with unpaired *t*-test. Spearman correlations between *ZC3H18* versus *BRCA1* and *E2F4* versus *BRCA1* mRNA expression in HGSOC PDX and patient tumors (Fig. 7a) were performed in R version 3.4.2 (https://www.R-project.org/). The following depicts statistical significance: *p < 0.05, **p < 0.01, ***p < 0.001.

### Reporting summary

Further information on research design is available in the Nature Research Reporting Summary linked to this article.

## Supplementary information

Supplementary Information

Description of Additional Supplementary Files

Supplementary Data 1

Supplementary Data 2

Reporting Summary

Source Data

## Data Availability

RNA-seq data have been deposited in the GEO accession GSE136533 and also provided as Supplementary Data 1 and 2. The authors declare that all other data supporting the findings of this study are available within the paper and its supplementary information and source data files upon reasonable request.
