# Supplementary material for: ZC3H18 specifically binds and activates the BRCA1 promoter to facilitate homologous recombination in ovarian cancer
Source: Nat Commun. 2019 Oct 11;10:4632. doi: 10.1038/s41467-019-12610-x (PMC6789141; doi:10.1038/s41467-019-12610-x)
Supplement: Supplementary file 1 — Supplementary Information [file 41467_2019_12610_MOESM1_ESM.pdf]

SUPPLEMENTARY INFORMATION

ZC3H18 Specifically Binds and Activates the BRCA1 Promoter to Facilitate Homologous Recombination in Ovarian Cancer

Kanakkanthara et al.

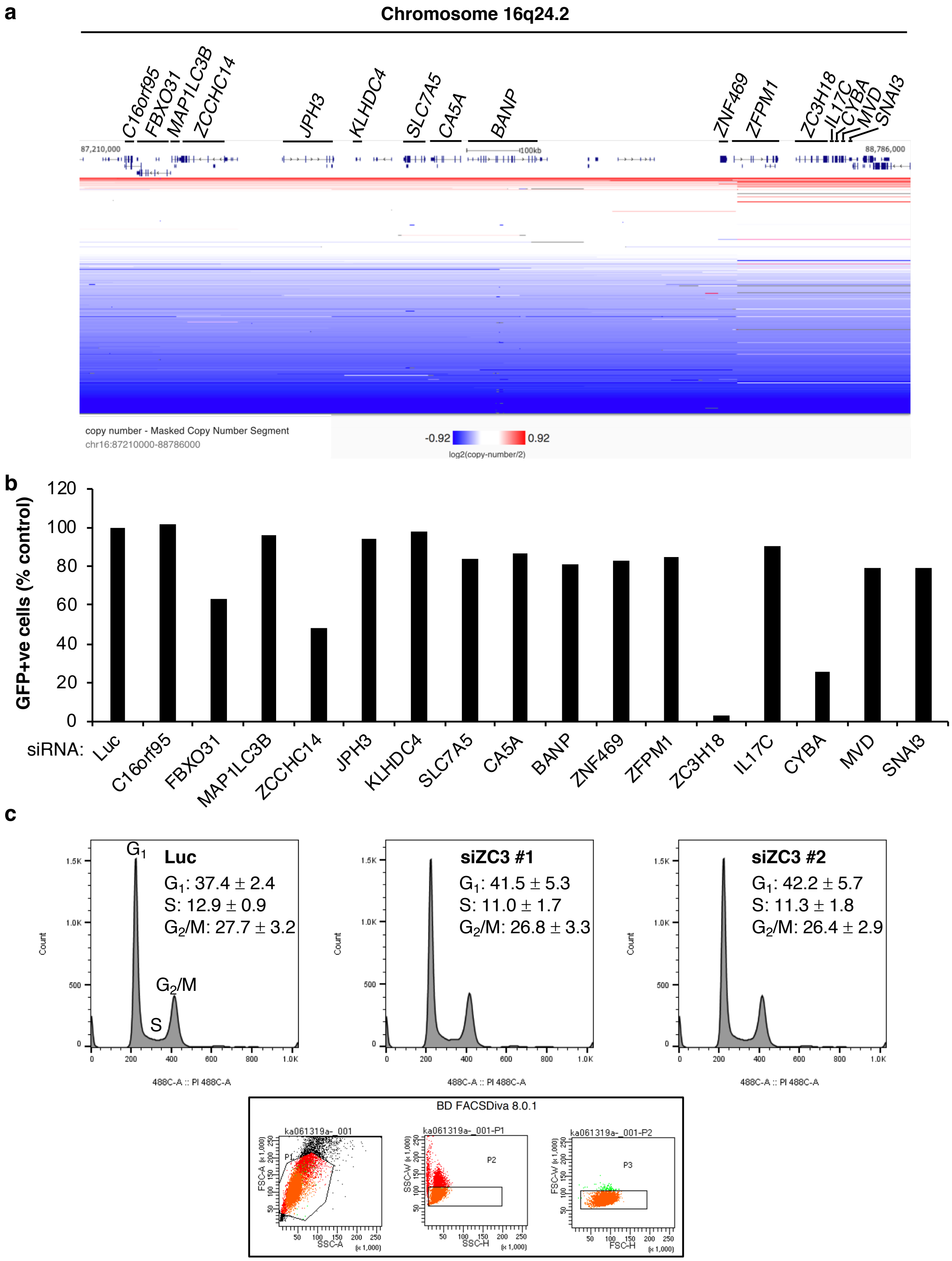

Supplementary Figure 1. (a) Heat map representation of chromosome 16q24.2 copy number changes in 587 HGSOC patients in the GDC TCGA Ovarian Cancer dataset analyzed using the

UCSC Xena browser (https://xenabrowser.net). The relative positions of the protein-coding genes in 16q24.2 are indicated. **(b)** Analysis of HR efficiency using OVCAR-8-DR-GFP cells transfected with the indicated siRNA pools that target the protein-coding genes on chromosome 16q24.2. Relative HR efficiency was normalized to cells transfected with Luc siRNA. **(c)** Cell cycle profiles of ZC3H18-depleted OVCAR-8 cells. OVCAR-8 cells were transfected with control luciferase (Luc) or ZC3H18 siRNAs. 48 hours later the cells were trypsinized, stained with propidium iodide, and analyzed by flow cytometry. Bottom panels show sequential gating strategy used for all cell cycle analyses. The data from the siRNA screen in (**b)** are from a single experiment. Representative images in (**c**) are from three independent experiments, and the means ± SEM of each cell cycle phase are presented in the image.

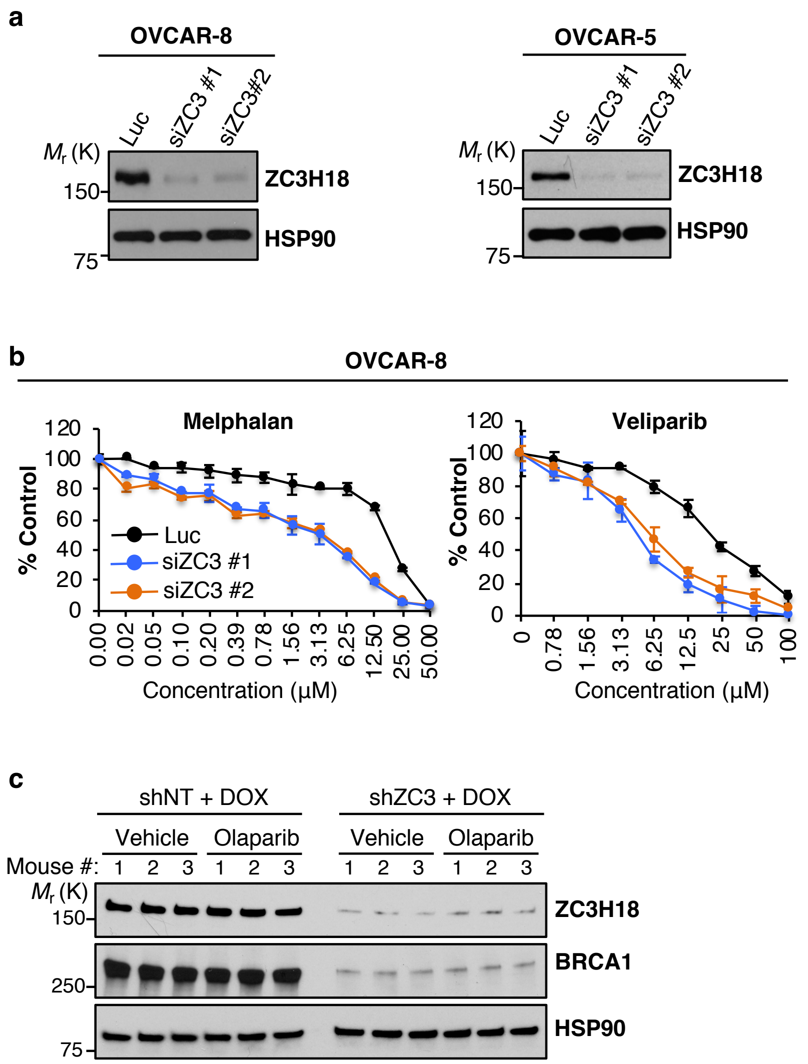

Supplementary Figure 2. (a) Immunoblots of ZC3H18 in OVCAR-8 and OVCAR-5 cells transfected with control luciferase (Luc) or two independent ZC3H18 siRNAs (**related to Fig. 1d** and **e**). **(b)** OVCAR-8 cells were transfected with control luciferase (Luc) or ZC3H18 siRNAs. 48 hours later the cells were trypsinized and processed for immunoblotting of ZC3H18 and HSP90 (loading control) (a) or were re-plated and treated with the indicated agents for 3 days (melphalan) or 7 days (veliparib), and analyzed by MTS assay. **(c)** Immunoblots of ZC3H18, BRCA1, and HSP90 (loading control) in xenografted OVCAR-8 cells with stably transduced, doxorubicin-inducible non-targeting shRNA (shNT) or ZC3H18 shRNA (shZC3) (**related to Fig. 1f**). Tumor tissues were collected after 4 weeks of vehicle or

olaparib treatment, and tumor tissues from three individual mice per treatment group were immunoblotted for ZC3H18, BRCA1 and HSP90 (loading control). The graphs in **b** represent one of three independent experiments that gave similar results. Immunoblots in **a** show results from a single experiment that are representative of three independent experiments. Data shown in **b** are means ± SEM from a single experiment that is representative of 3 independent experiments. Immunoblots in **c** are from three independent mice per treatment group from a single experiment. Unprocessed blots are provided in Source data file.

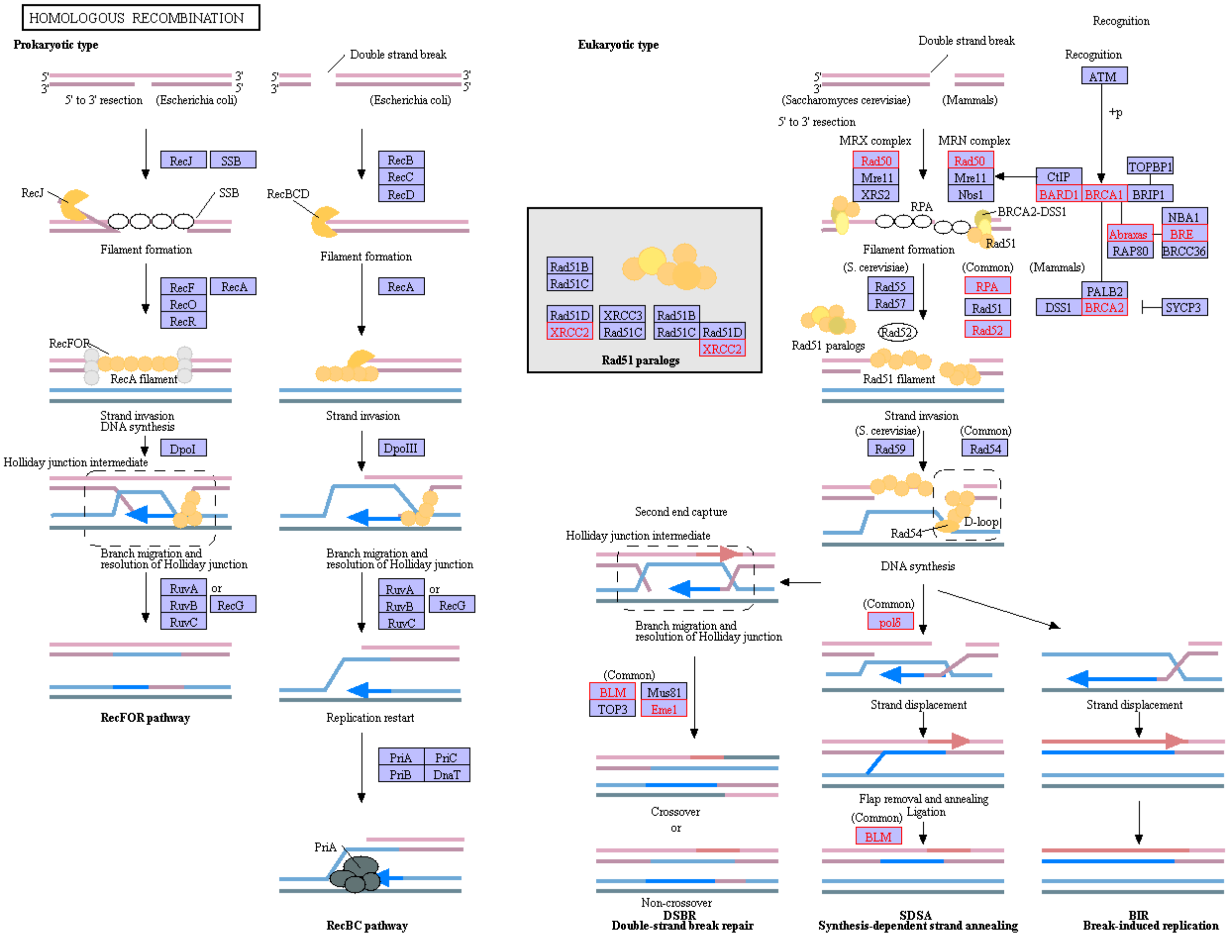

Supplementary Figure 3. KEGG Pathway analysis for HR genes differentially expressed in the RNA-seq (Supplementary Table 1) of ZC3H18 siRNA-transfected OVCAR-8 cells compared to control luciferase (Luc) siRNA-transfected cells. HR-associated genes with FDR < 0.05 are shown in red. RNA-seq analysis was performed from cells transfected with two separate siRNAs targeting ZC3H18 and using 3 independent samples for each siRNA.

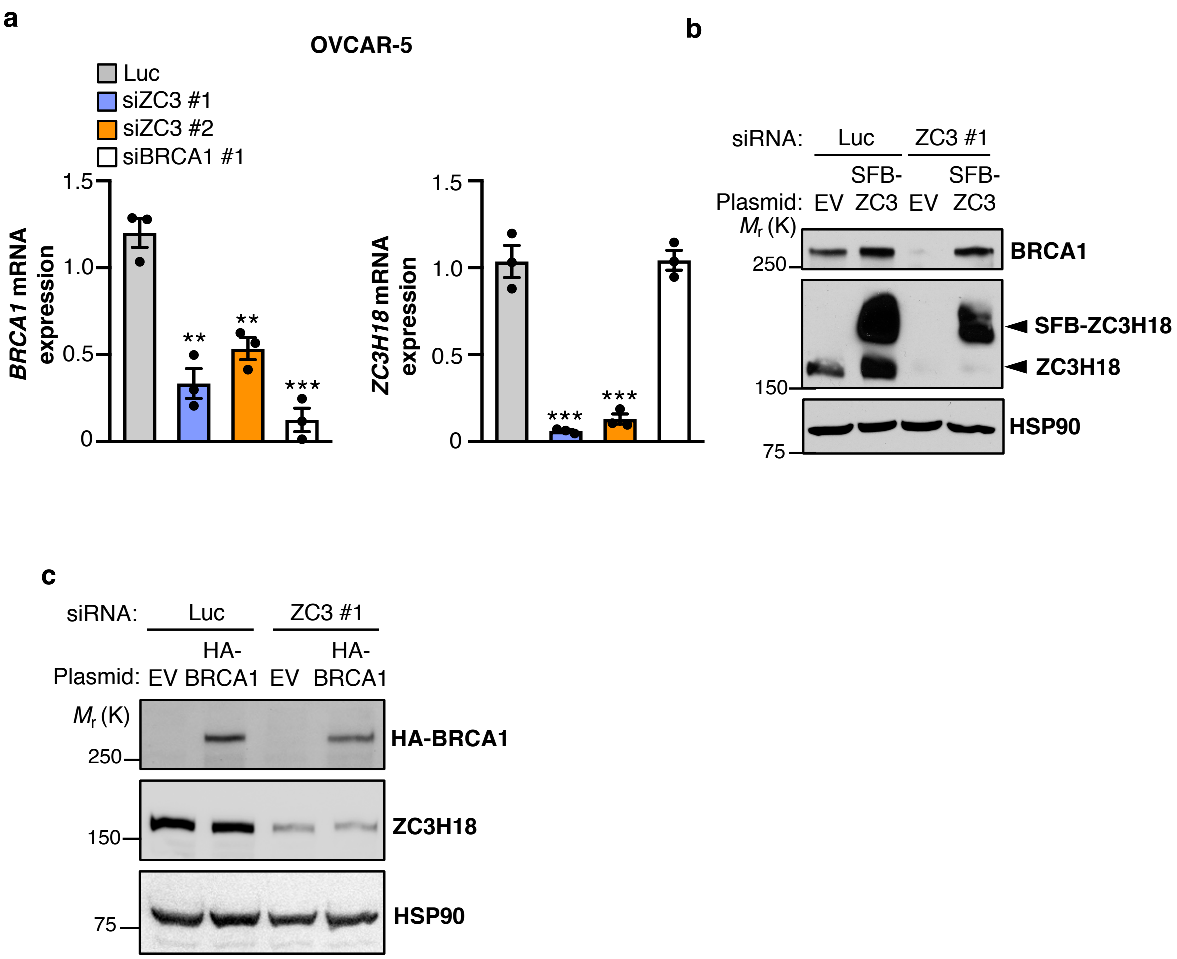

Supplementary Figure 4. (a) *BRCA1* and *ZC3H18* mRNA levels in ZC3H18 or BRCA1 siRNA-transfected OVCAR-5 cells determined by qRT-PCR. The mRNA levels are expressed as relative to *GAPDH* mRNA as the internal control. **(b)** OVCAR-8 cells were transfected with Luc or ZC3H18 siRNA plus empty vector (EV) or siRNA-resistant ZC3H18 plasmid (SFB-ZC3H18). BRCA1 and ZC3H18 proteins levels were assessed by immunoblotting (**related to Fig. 2c**). **(c)** OCVAR-8 DR-GFP cells were transfected with empty vector (EV) or HA-BRCA1-expressing plasmid with either control luciferase (Luc) or ZC3H18 (ZC3) siRNAs. Seventy-two hours later, HA-BRCA1 and ZC3H18 levels were examined by immunoblotting. HSP90 was used as loading control (**related to Fig. 2d**). Data shown in **a** are means ± SEM from 3 independent experiments.

$p < 0.01$, ***$p < 0.001$, unpaired Student's *t* test. Representative immunoblots in **b and **c** are from three independent experiments. Unprocessed blots are provided in Source data file.

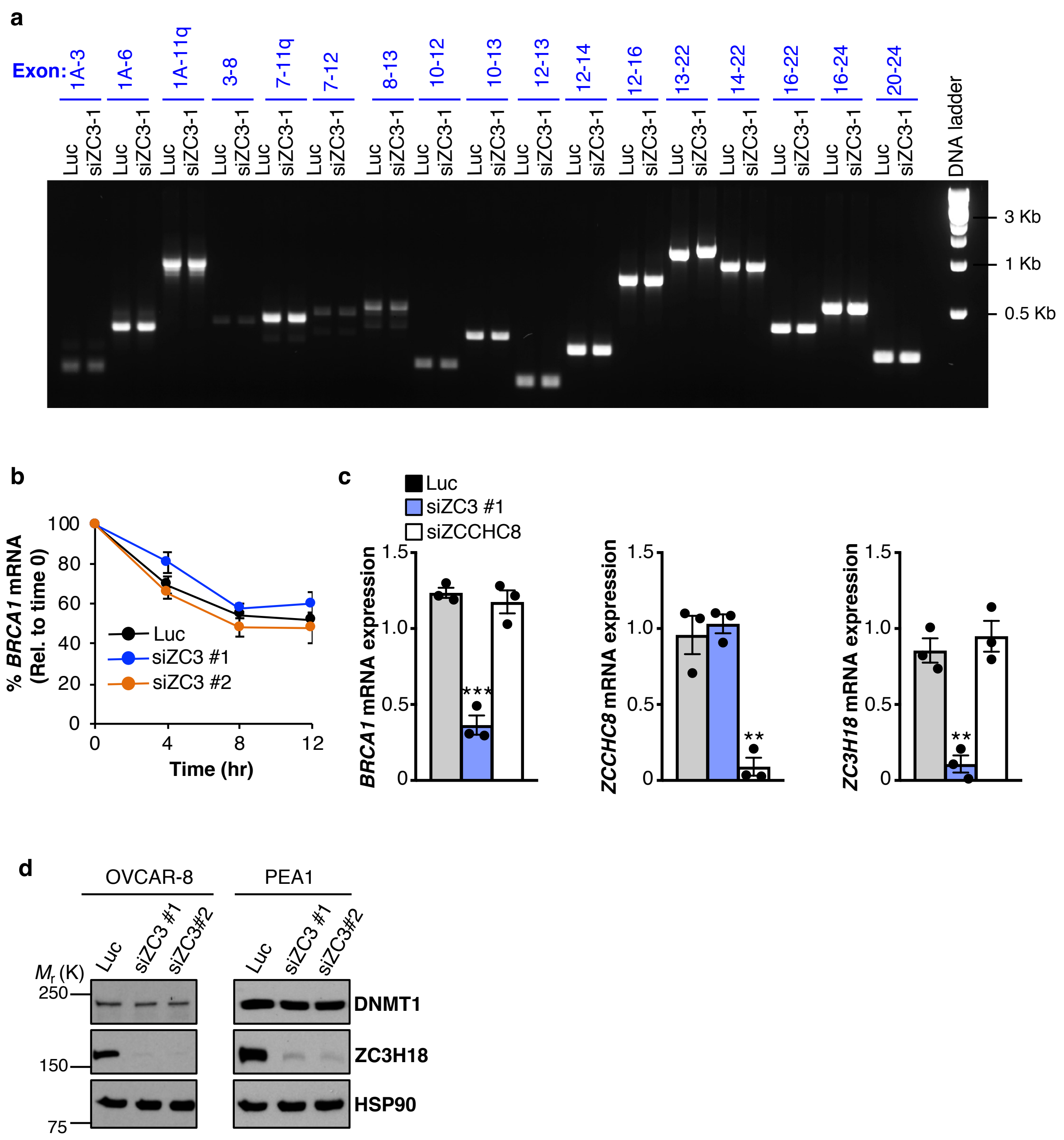

Supplementary Figure 5. (a) *BRCA1* alternative splicing events, in control (Luc) or ZC3H18 siRNA-transfected OVCAR-8 cells determined by PCR using multiple combinations of forward and reverse primers located in *BRCA1* exonic regions. **(b)** *BRCA1* mRNA stability in ZC3H18-depleted cells. OVCAR-8 cells were transfected with control (Luc) siRNA or two-independent ZC3H18 siRNAs, and 48 hours after transfection, the cells were treated with the transcription

inhibitor actinomycin D (5 μg/ml) for indicated time periods. *BRCA1* mRNA levels, normalized to *GAPDH* mRNA levels, were determined by qRT-PCR. **(c)** *BRCA1, ZCCHC8,* and *ZC3H18* mRNA levels, normalized to *GAPDH* mRNA expression, were determined by qRT-PCR in OVCAR-8 cells transfected with ZCCHC8 or ZC3H18 siRNAs. **(d)** Immunoblots of indicated proteins in control and ZC3H18-depleted OVCAR-8 and PEA1 cells (**related to Fig. 3b**). Images in **a** and **d** and graph in **b** are representative of three independent experiments that gave similar results. Data in **c** are means ± SEM from 3 independent experiments. $^{**}p < 0.01$, $^{***}p < 0.001$, unpaired Student's *t* test. Unprocessed blots of **d** are provided in Source data file.

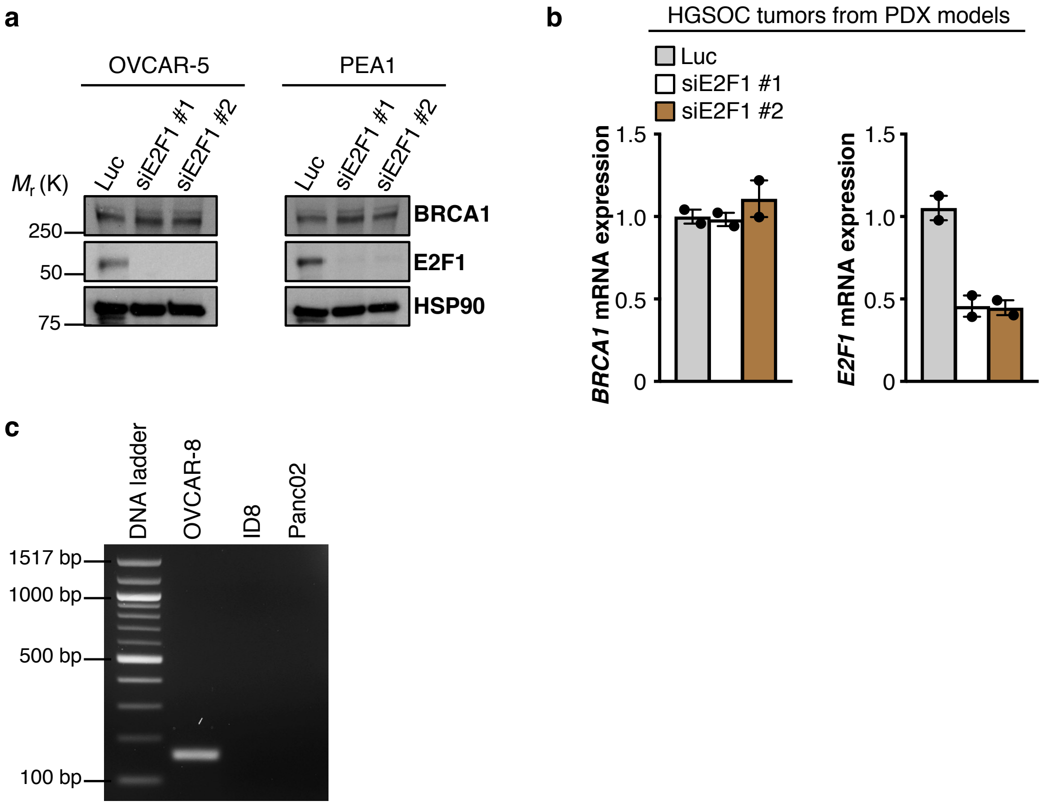

Supplementary Figure 6. (a) Immunoblots of indicated proteins in control luciferase (Luc) and E2F1 siRNA-transfected OVCAR-5 and PEA1 cells (**related to Fig. 4c-d**). **(b)** *BRCA1* mRNA expression in short-term *ex vivo* cultures of HGSOC tumors from two different PDX models transfected with Luc or E2F1 siRNAs. **(c)** Agarose gel electrophoresis demonstrating that the BRCA1 qPCR primers used in this study are specific for the human *BRCA1* transcript. Total RNA was extracted and cDNA was synthesized from OVCAR-8 cells, mouse pancreatic ductal adenocarcinoma Panc-02 cells, and mouse ovarian surface epithelium ID-8 cells, and qPCR was performed using the conditions described in the method section. Images in **a** are representative of three independent experiments that gave similar results. Unprocessed blots of **a** are provided in Source data file. Data in **b** are means ± SEM from two independent PDX models. Image in **c** is from one experiment.

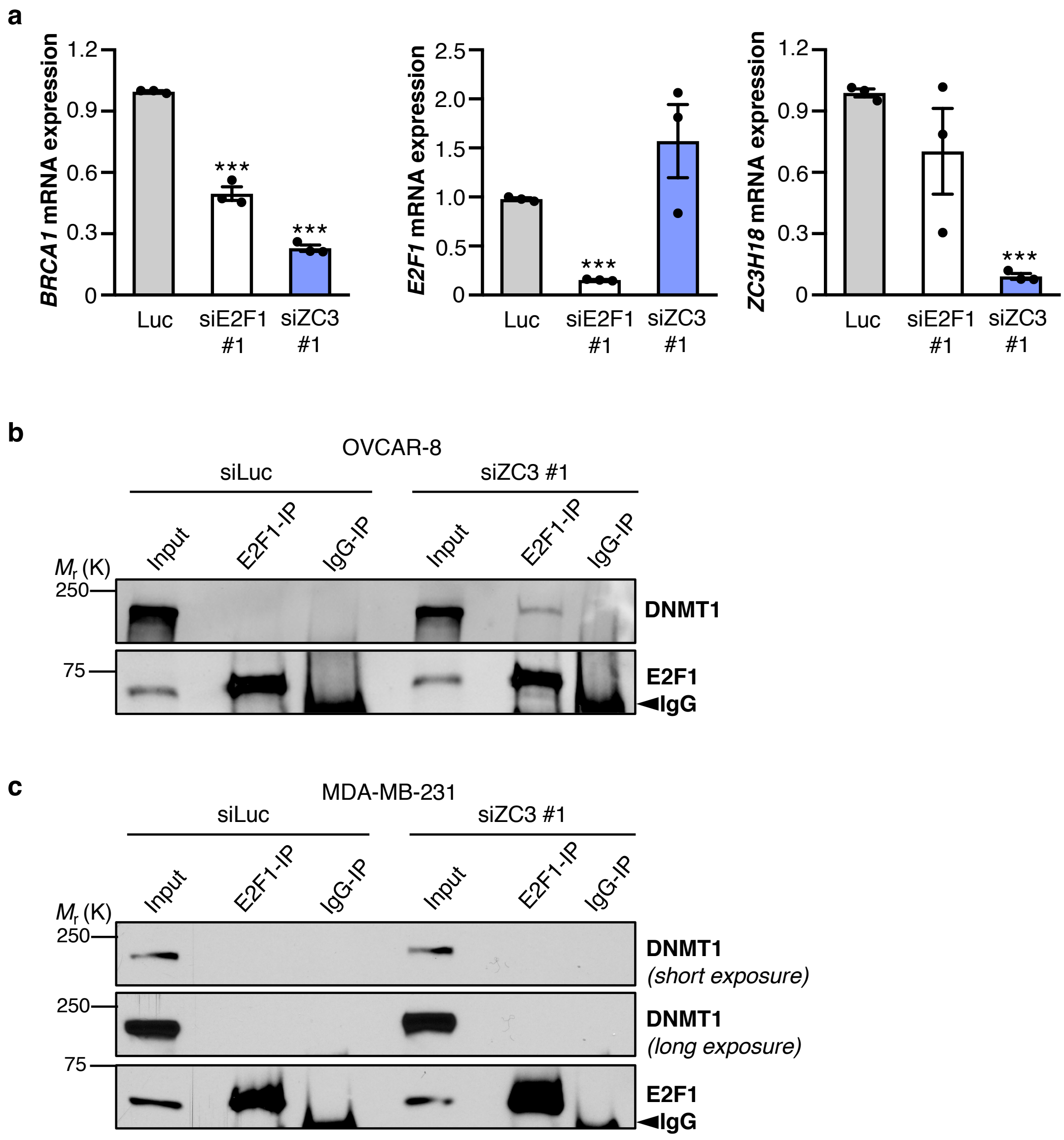

Supplementary Figure 7. (a) *BRCA1*, *E2F1,* and *ZC3H18* mRNA levels, normalized to *GAPDH* mRNA expression, were determined by qRT-PCR in MDA-MB-231 cells transfected with control luciferase (Luc) or E2F1 siRNAs. (**b** and **c**) OVCAR-8 (**b**) or MDA-MB-231 (**c**) cells were transfected with control luciferase (Luc) or ZC3H18 siRNAs. After 48 hours, cells lysates were immunoprecipitated with an E2F1 antibody, and the immunoprecipitates were immunoblotted for DNMT1. Data in **a** are means ± SEM from three independent experiments. ***$p <$ 0.001, unpaired Student's *t* test compared to Luc-transfected cells. Representative

immunoblots in **b** and **c** are from three independent experiments. Unprocessed blots are provided in Source data file.

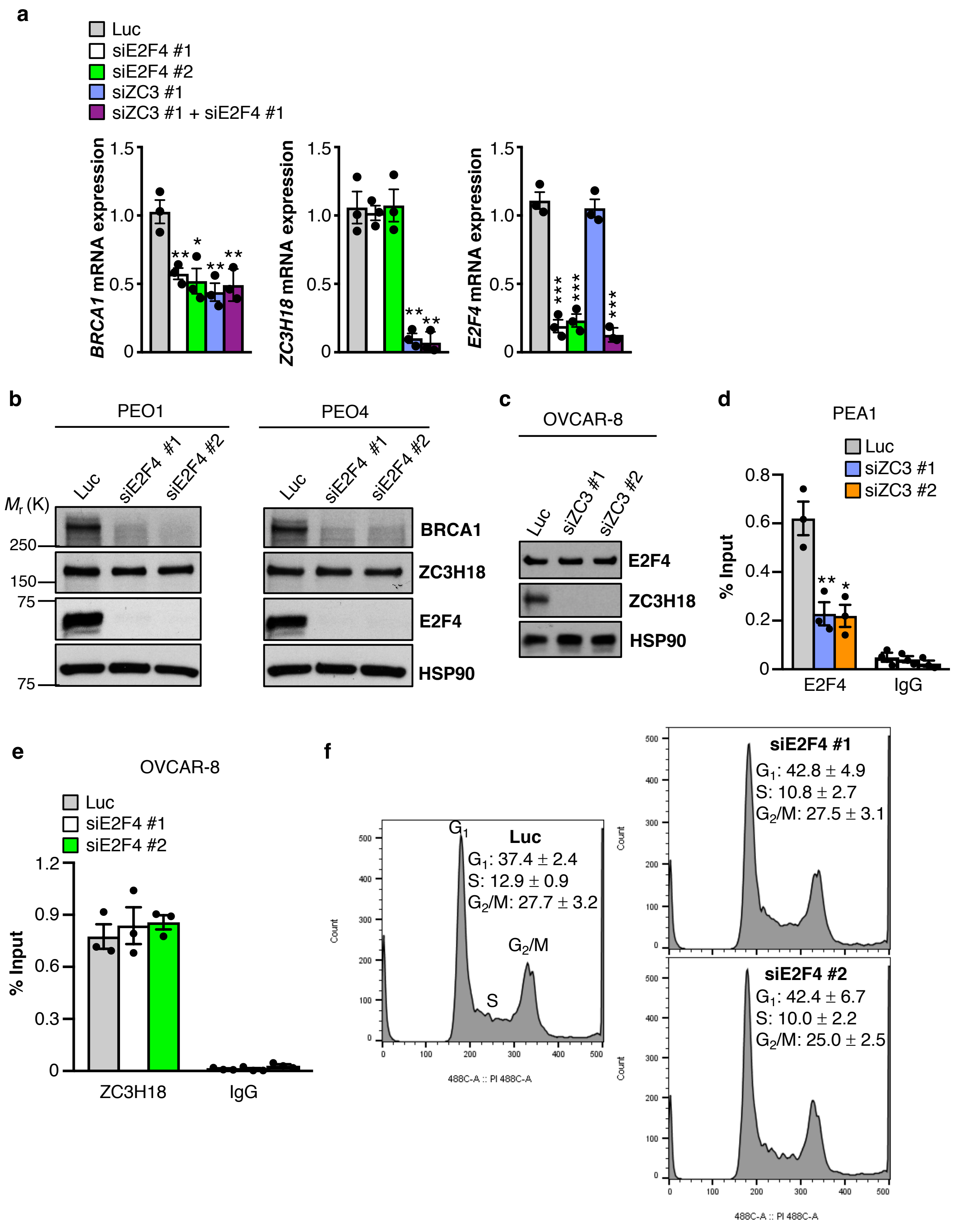

Supplementary Figure 8. (a) *BRCA1*, *ZC3H18,* and *E2F4* mRNA levels, normalized to *GAPDH* mRNA expression, were determined by qRT-PCR in OVCAR-8 cells transfected with the

indicated siRNAs. **(b)** Immunoblots of indicated proteins in E2F4 siRNA-transfected PEO1 and PEO4 cells. **(c)** Immunoblots of indicated proteins in ZC3H18 siRNA-transfected OVCAR-8 cells (**related to Fig. 5d**). **(d)** ChIP assay showing E2F4 occupancy on the *BRCA1* promoter in ZC3H18 siRNA-transfected PEA1 cells using anti-E2F4 or IgG control antibodies and primers specific for the *BRCA1* promoter. **(e)** ChIP assay showing ZC3H18 occupancy on the *BRCA1* promoter in E2F4 siRNA-transfected OVCAR-8 cells. **(f)** Cell cycle profile of E2F4-depleted OVCAR-8 cells. OVCAR-8 cells were transfected with control luciferase (Luc) or two independent E2F4 siRNAs. 48 hours later, the cells were trypsinized, stained with propidium iodide, and analyzed by flow cytometry. Data in **a**, **d**, and **e** are means ± SEM from three independent experiments. $^{**}p < 0.01$, $^{***}p < 0.001$, unpaired Student's *t* test compared to Luc-transfected samples. Representative immunoblots in **b** and **c** are from three independent experiments. Unprocessed blots are provided in Source data file. Representative images in **f** are from three independent experiments, and the means ± SEM of each cell cycle phase are presented in the image. The cell cycle data shown for control luciferase (Luc) siRNA-transfected cells are the same as shown in Supplementary Fig. 1a because the studies to assess the impact of depleting ZC3H18 and E2F4 were done in the same experiments.

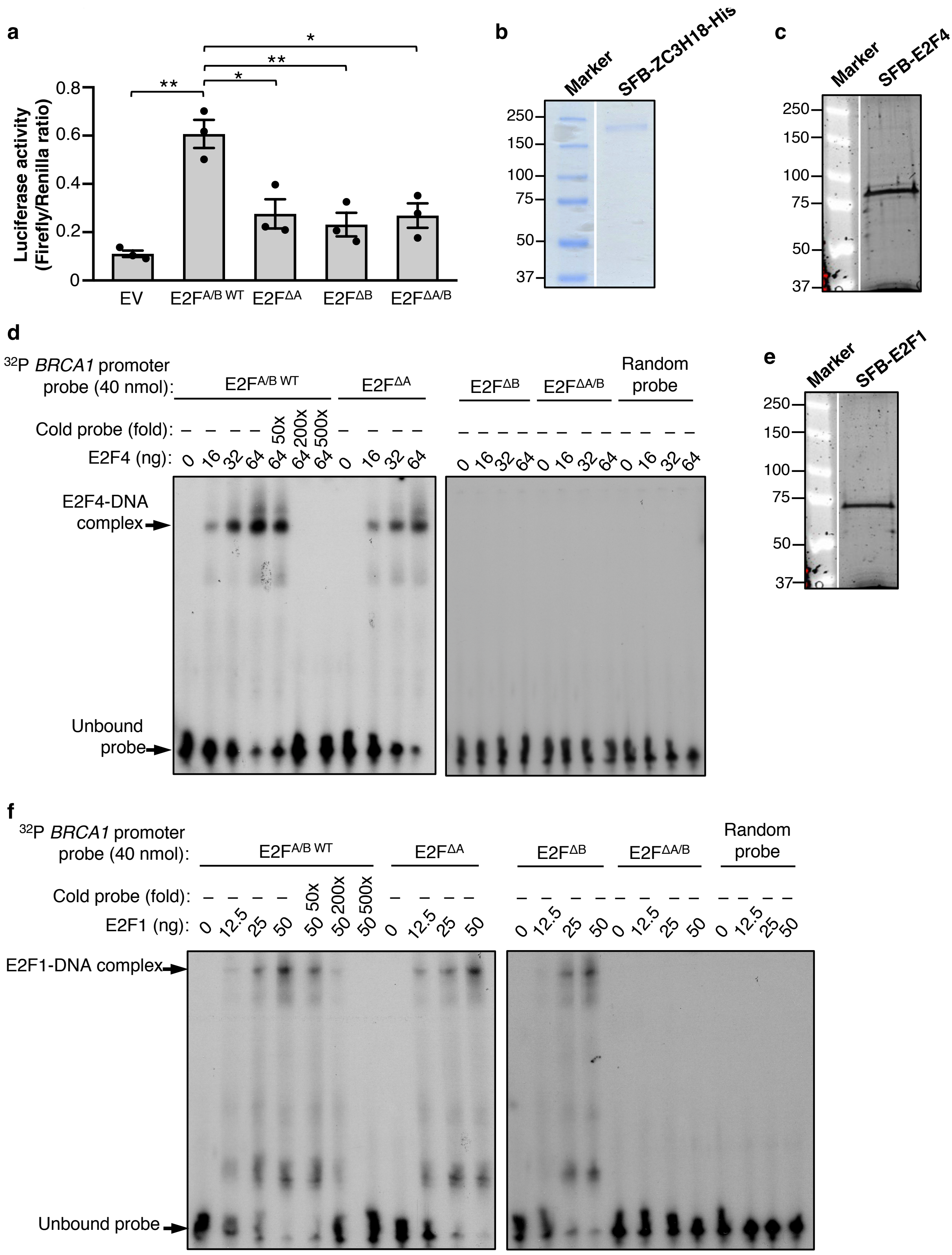

Supplementary Figure 9. (a) Luciferase reporter assay using OVCAR-8 cells transfected with

BRCA1 luciferase promoter pBRC-FF constructs with wild-type sequence (E2FA/B^{WT}) and mutated E2FA (E2$F^{\Delta A}$), E2FB (E2$F^{\Delta B}$), and E2FA and E2FB (E2$F^{\Delta A/B}$) sites.

Renilla luciferase reporter plasmid was used as internal control. **(b)** Coomassie Blue-stained SDS-PAGE gel showing recombinant SFB-ZC3H18-His purified from *E. coli*. **(c)** SYPRO® Ruby-stained SDS-PAGE gel showing purified SFB-E2F4. **(d)** EMSA with purified SFB-E2F4 using wild-type BRCA1 (E2FA/B^{WT}) and mutated E2FA (E2$F^{\Delta A}$), E2FB (E2$F^{\Delta B}$), E2FA and E2FB (E2$F^{\Delta A/B}$) sites promoter probes. A probe with randomly shuffled sequence was used as negative control. **(e)** SYPRO® Ruby-stained SDS-PAGE gel showing purified SFB-E2F1. **(f)** Same as in **d**, but the EMSA was done with purified SFB-E2F1. All EMSA images are representative of 3 independent experiments that gave similar results. Data in **a** are means ± SEM from 3 independent experiments. *$p < 0.05$, **$p < 0.01$, unpaired Student's *t* test.

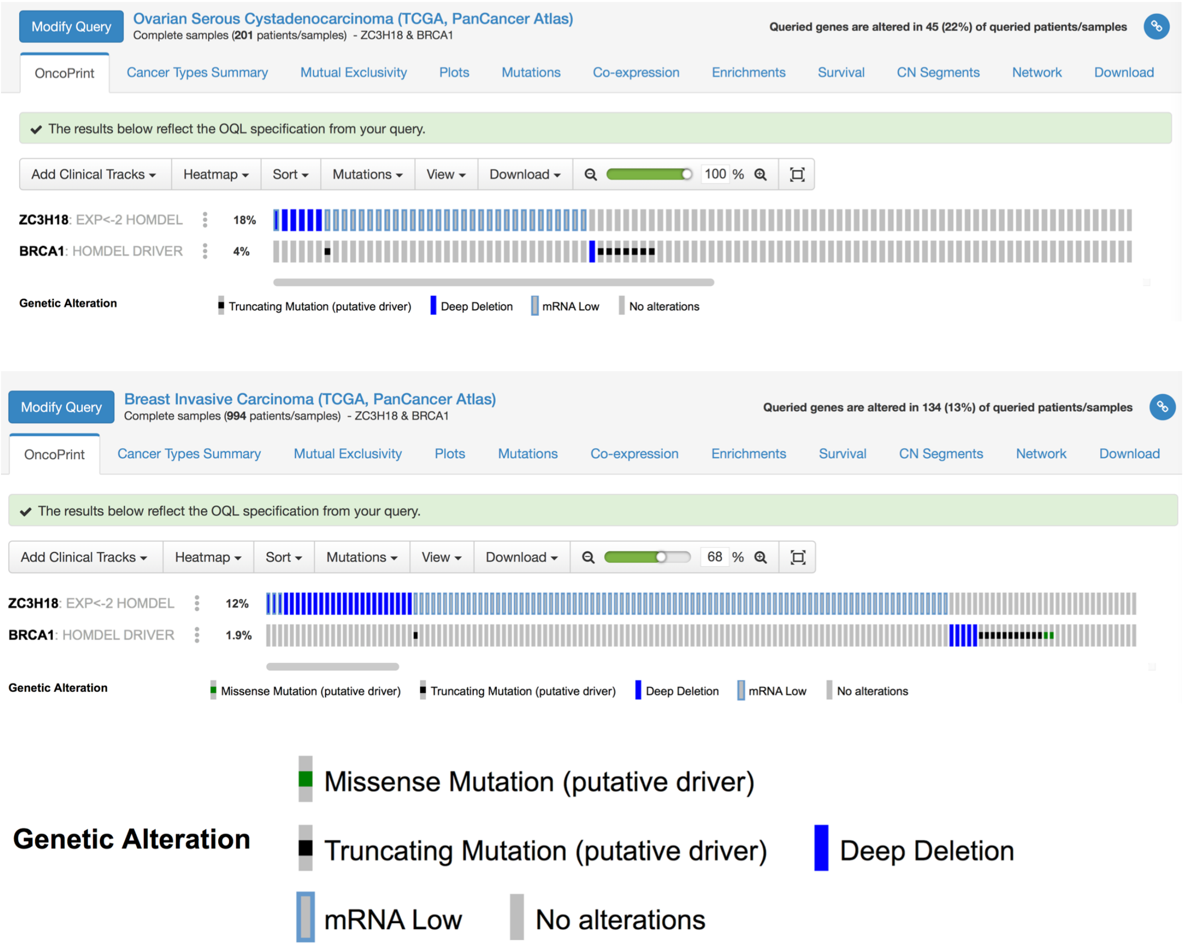

Supplementary Figure 10. Oncoprints generated in cBioPortal showing deep (homologous) deletions and mRNA expression < 2 standard deviations below mean for *ZC3H18* and deep (homologous) deletions and driver mutations for *BRCA1* in ovarian serous cystadenocarcinoma (https://www.cbioportal.org/results/oncoprint?session_id=5d25e9e1e4b0ab413787a939) and breast invasive carcinoma (https://www.cbioportal.org/results/oncoprint?session_id=5d25eaf4e4b0ab413787a945) samples in the PanCancer Atlas Databases.
