# Supplementary material for: ZC3H18 specifically binds and activates the BRCA1 promoter to facilitate homologous recombination in ovarian cancer
Source: Nat Commun. 2019 Oct 11;10:4632. doi: 10.1038/s41467-019-12610-x (PMC6789141; doi:10.1038/s41467-019-12610-x)
Supplement: Supplementary file 2 — Description of Additional Supplementary Files [file 41467_2019_12610_MOESM2_ESM.pdf]

Description of Additional Supplementary Files

File Name: Supplementary Data 1
Description: Differential gene expression in OVCAR-8 cells transfected with control luciferase (Luc) vs. ZC3H18 (ZC3) siRNAs.

File Name: Supplementary Data 2
Description: ZC3H18, BRCA1, and E2F4 mRNA expression levels in patient and PDX HGSOC tumors.
