# Supplementary material for: ZC3H18 specifically binds and activates the BRCA1 promoter to facilitate homologous recombination in ovarian cancer
Source: Nat Commun. 2019 Oct 11;10:4632. doi: 10.1038/s41467-019-12610-x (PMC6789141; doi:10.1038/s41467-019-12610-x)
Supplement: Supplementary file 5 — Reporting Summary [file 41467_2019_12610_MOESM5_ESM.pdf]

natureresearch

Corresponding author(s): NCOMMS-18-23827-T
Larry Karnitz

Last updated by author(s): Aug 30, 2019

Reporting Summary

Nature Research wishes to improve the reproducibility of the work that we publish. This form provides structure for consistency and transparency in reporting. For further information on Nature Research policies, see Authors & Referees and the Editorial Policy Checklist.

Statistics

For all statistical analyses, confirm that the following items are present in the figure legend, table legend, main text, or Methods section.

n/a	Confirmed	
☐	☒	The exact sample size (*n*) for each experimental group/condition, given as a discrete number and unit of measurement
☐	☒	A statement on whether measurements were taken from distinct samples or whether the same sample was measured repeatedly
☐	☒	The statistical test(s) used AND whether they are one- or two-sided<br>*Only common tests should be described solely by name; describe more complex techniques in the Methods section.*
☐	☒	A description of all covariates tested
☐	☒	A description of any assumptions or corrections, such as tests of normality and adjustment for multiple comparisons
☐	☒	A full description of the statistical parameters including central tendency (e.g. means) or other basic estimates (e.g. regression coefficient) AND variation (e.g. standard deviation) or associated estimates of uncertainty (e.g. confidence intervals)
☐	☒	For null hypothesis testing, the test statistic (e.g. *F*, *t*, *r*) with confidence intervals, effect sizes, degrees of freedom and *P* value noted<br>*Give P values as exact values whenever suitable.*
☒	☐	For Bayesian analysis, information on the choice of priors and Markov chain Monte Carlo settings
☒	☐	For hierarchical and complex designs, identification of the appropriate level for tests and full reporting of outcomes
☐	☒	Estimates of effect sizes (e.g. Cohen's *d*, Pearson's *r*), indicating how they were calculated

Our web collection on statistics for biologists contains articles on many of the points above.

Software and code

Policy information about availability of computer code

Data collection	No software used.
Data analysis	The RNA-seq analyses of the OVCAR-8 cells was performed using MAP-RSeq.The MAP-RSeq software provides gene counts, exon counts, fusion candidates, expressed single nucleotide variants, mapping statistics, visualizations, and a detailed research data report for RNA-Seq experiments. The edgeR software examines gene count data from RNA-Seq experiments to quantify differential expression.<br><br>The ovarian cancer PDX RNA-seq analyses were performed using R version 3.4.2 (https://www.R-project.org/).

For manuscripts utilizing custom algorithms or software that are central to the research but not yet described in published literature, software must be made available to editors/reviewers. We strongly encourage code deposition in a community repository (e.g. GitHub). See the Nature Research guidelines for submitting code & software for further information.

Data

Policy information about availability of data

All manuscripts must include a data availability statement. This statement should provide the following information, where applicable:

- Accession codes, unique identifiers, or web links for publicly available datasets
- A list of figures that have associated raw data
- A description of any restrictions on data availability

RNA-seq data have been deposited in the GEO accession GSE136533 [https://www.ncbi.nlm.nih.gov/geo/query/acc.cgi?acc=GSE136533] and also provided as Supplementary Data File 1 and 2. The authors declare that all other data supporting the findings of this study are available within the paper and its supplementary information and source data files upon reasonable request.

Field-specific reporting

Please select the one below that is the best fit for your research. If you are not sure, read the appropriate sections before making your selection.

☒ Life sciences ☐ Behavioural & social sciences ☐ Ecological, evolutionary & environmental sciences

For a reference copy of the document with all sections, see nature.com/documents/nr-reporting-summary-flat.pdf

Life sciences study design

All studies must disclose on these points even when the disclosure is negative.

Sample size	For in vitro experiments, we used 3 or more replicates based on previous experience with this studies. For the xenograft studies 10 mice per treatment group were used based on our previous experience with these assays. For the RNA-seq analyses of primary human tumors and PDX tumors, we used all the available data (97 patient tumors, 138 PDX tumors). For the comparison of ZC3H18 copy number and BRCA1 mutations in HGSOC and breast cancers, we used all the data available in the TCGA database (489 HGSOC samples, 825 breast cancer samples).
Data exclusions	No data were excluded from the analyses.
Replication	All in vitro experiments were replicated a minimum of 3 times (n = 3). The in vivo xenograft studies were performed once with 10 mice per treatment group. The Western blots from the in vivo xenograft tumors were performed once. The RNA-seq experiment was performed once with 3 biological replicates per siRNA.
Randomization	For the in vivo xenograft studies, mice were randomized before treatment with 10 mice per treatment group.
Blinding	No blinding was performed.

Reporting for specific materials, systems and methods

We require information from authors about some types of materials, experimental systems and methods used in many studies. Here, indicate whether each material, system or method listed is relevant to your study. If you are not sure if a list item applies to your research, read the appropriate section before selecting a response.

Materials & experimental systems

n/a	Involved in the study	
☐	☒	Antibodies
☐	☒	Eukaryotic cell lines
☒	☐	Palaeontology
☐	☒	Animals and other organisms
☐	☒	Human research participants
☒	☐	Clinical data

Methods

n/a	Involved in the study	
☒	☐	ChIP-seq
☐	☒	Flow cytometry
☒	☐	MRI-based neuroimaging

Antibodies

Antibodies used

RAD51 (1:250, PC-130, Calbiochem)
phospho-histone H2A.X (γ-H2A.X) (1:250, 05-636, Millipore)
ZC3H18 (1:1000, A304-682A, Bethyl Laboratories Inc.)
BRCA1 (1:2000, sc-6954, Santa Cruz Biotechnology
E2F1 (1:500, ab4070, Abcam)
E2F4 (1:1000, NBP1-21374, Novus Biologicals
DNMT1 (1:5000, ab13537, Abcam)
HSP90 (D. Toft, Mayo Clinic, H9010).
anti-mouse immunoglobulin G horseradish peroxidase-conjugated (1:2000 for BRCA1 and 1:10,000 for all other primary antibodies, 7076S, Cell Signaling Technology

anti-rabbit immunoglobulin G horseradish peroxidase-conjugated (1:16,000 for ZC3H18 and 1:10,000 for all other primary antibodies, 7074S, Cell Signaling Technology).

anti-rabbit Alexa Fluor® 488–conjugated secondary antibody (1:500; ThermoFisher Scientific)
anti-mouse Alexa Fluor® 594–conjugated secondary antibody (1:500; ThermoFisher Scientific)

normal mouse IgG (1 μg/ChIP, 0107-01, SouthernBiotech)
normal rabbit IgG (1 μg/ChIP, 0111-01, SouthernBiotech)

	anti-FLAG M2 antibody (F3165, Sigma)<br><br>anti-S-tag antibody (Biotechniques. 2004 Nov;37(5):835-9).
Validation	All antibodies were validated by showing that at least 2 siRNAs depleted an immunoreactive band of the correct size.

Eukaryotic cell lines

Policy information about cell lines

Cell line source(s)	OVCAR-8, D. Scuderio (National Cancer Institute)<br>OVCAR-5, D. Scuderio (National Cancer Institute)<br>PEA1, Sigma-Aldrich<br>PEO4, T. Taniguchi (Fred Hutchinson Cancer Research Institute)
Authentication	All cell lines were authenticated by autosomal STR profiling using a service at the University of Arizona.
Mycoplasma contamination	All cell lines were tested for mycoplasma contamination.
Commonly misidentified lines (See ICLAC register)	No commonly misidentified cell lines were used.

Animals and other organisms

Policy information about studies involving animals; ARRIVE guidelines recommended for reporting animal research

Laboratory animals	8-week-old SCID Beige female mice C.B-17/IcrHsd-Prkdcscid Lystbg-J were used.
Wild animals	No wild animals were used.
Field-collected samples	No field-collected samples were used.
Ethics oversight	All studies with animals were approved by the Mayo Clinic Institutional Animal Care and Use Committee.

Note that full information on the approval of the study protocol must also be provided in the manuscript.

Human research participants

Policy information about studies involving human research participants

Population characteristics	All patients were at least 18 yrs of age, female, and diagnosed with ovarian, fallopian tube, or primary peritoneal cancer. All tumors specimens were collected at the time of initial surgical debulking at Mayo Clinic.
Recruitment	Prior to surgical resection of the primary tumor(s), patients are seen in the clinic to discuss risks/benefits of participation in the Mayo Clinic ovarian tumor repository. All patients were approached without bias toward baseline characteristics such as, but not limited to, age, ethnicity, genetic/familial predisposition for cancer, tumor stage, tumor grade, or histology. Patients may have declined participation for personal reasons, which were independent of the study team. Accordingly, patient selection was unlikely to impact results.
Ethics oversight	The studies were conducted under the aegis of approval by the Mayo Clinic Institutional Review Board.

Note that full information on the approval of the study protocol must also be provided in the manuscript.

Flow Cytometry

Plots

Confirm that:

☒ The axis labels state the marker and fluorochrome used (e.g. CD4-FITC).

☒ The axis scales are clearly visible. Include numbers along axes only for bottom left plot of group (a 'group' is an analysis of identical markers).

☒ All plots are contour plots with outliers or pseudocolor plots.

☒ A numerical value for number of cells or percentage (with statistics) is provided.

Methodology

Sample preparation	OVCAR-8 cells were transfected with siRNAs. 48 hours later, the cells were washed in PBS, fixed in 70% ethanol in PBS, rehydrated, treated with RNAse A, stained with propidium iodide, and analyzed.

Instrument	BD FACSCanto II
Software	BD FACS diva and analyzed using FlowJo to analyze cell cycle.
Cell population abundance	Cells were not sorted, only whole populations were analyzed.
Gating strategy	Based on FSC-A and SSC-A scatter, debris was excluded from the analysis; To remove doublets, we gated on SSC-W vs SSC-H, followed by FSC-W vs FSC-H.

☒ Tick this box to confirm that a figure exemplifying the gating strategy is provided in the Supplementary Information.
